# mRNA Therapeutics Beyond Infectious Diseases: Expanding Therapeutic Applications and Future Perspectives

**DOI:** 10.1002/iid3.70511

**Published:** 2026-09-21

**Authors:** Gedion Mengistu Dejen

**Affiliations:** ^1^ Department of Biotechnology Arba Minch University Arba Minch South Ethiopia Regional State Ethiopia

**Keywords:** cancer immunotherapy, lipid nanoparticles, messenger RNA, mRNA therapeutics, personalized medicine, precision medicine, regenerative medicine, translational medicine

## Abstract

**Background:**

Messenger RNA (mRNA) therapeutics have rapidly evolved from experimental nucleic acid constructs into a clinically validated therapeutic platform. Their successful application during the COVID‐19 pandemic accelerated interest in broader therapeutic uses beyond infectious diseases.

**Aims:**

This review summarizes the expanding therapeutic applications of mRNA technologies, recent technological advances, translational progress, current challenges, and future prospects across diverse medical fields.

**Materials and Methods:**

A comprehensive review of the published literature was conducted, synthesizing evidence from preclinical investigations, clinical studies, and recent advances in mRNA engineering, delivery systems, and translational research.

**Results:**

Advances in mRNA design, nucleoside modification, codon optimization, and delivery platforms, particularly lipid nanoparticles and emerging organ‐targeted carriers, have expanded applications to cancer immunotherapy, personalized vaccines, autoimmune and inflammatory diseases, protein replacement therapy, cardiovascular regeneration, neurological disorders, and rare genetic diseases. Emerging technologies, including self‐amplifying and circular RNA systems, further enhance therapeutic potential.

**Discussion:**

Despite significant progress, challenges remain, including optimization of targeted delivery, long‐term safety, repeat‐dose tolerability, manufacturing complexity, cold‐chain requirements, production costs, and equitable global access. Continued technological innovation and regulatory harmonization are critical for successful clinical translation.

**Conclusion:**

mRNA therapeutics are transitioning from a vaccine‐centered technology to a versatile platform for precision medicine. Advances in delivery systems, scalable manufacturing, and artificial intelligence‐assisted sequence design are expected to accelerate their integration into the treatment of chronic, genetic, and regenerative diseases.

## Graphical abstract

Messenger RNA therapeutics are rapidly expanding beyond infectious diseases into oncology, autoimmune disorders, protein replacement therapy, cardiovascular and neurological diseases, and regenerative medicine. Advances in RNA engineering and targeted delivery are driving the development of safe, programmable, and precision therapeutics for diverse clinical applications.

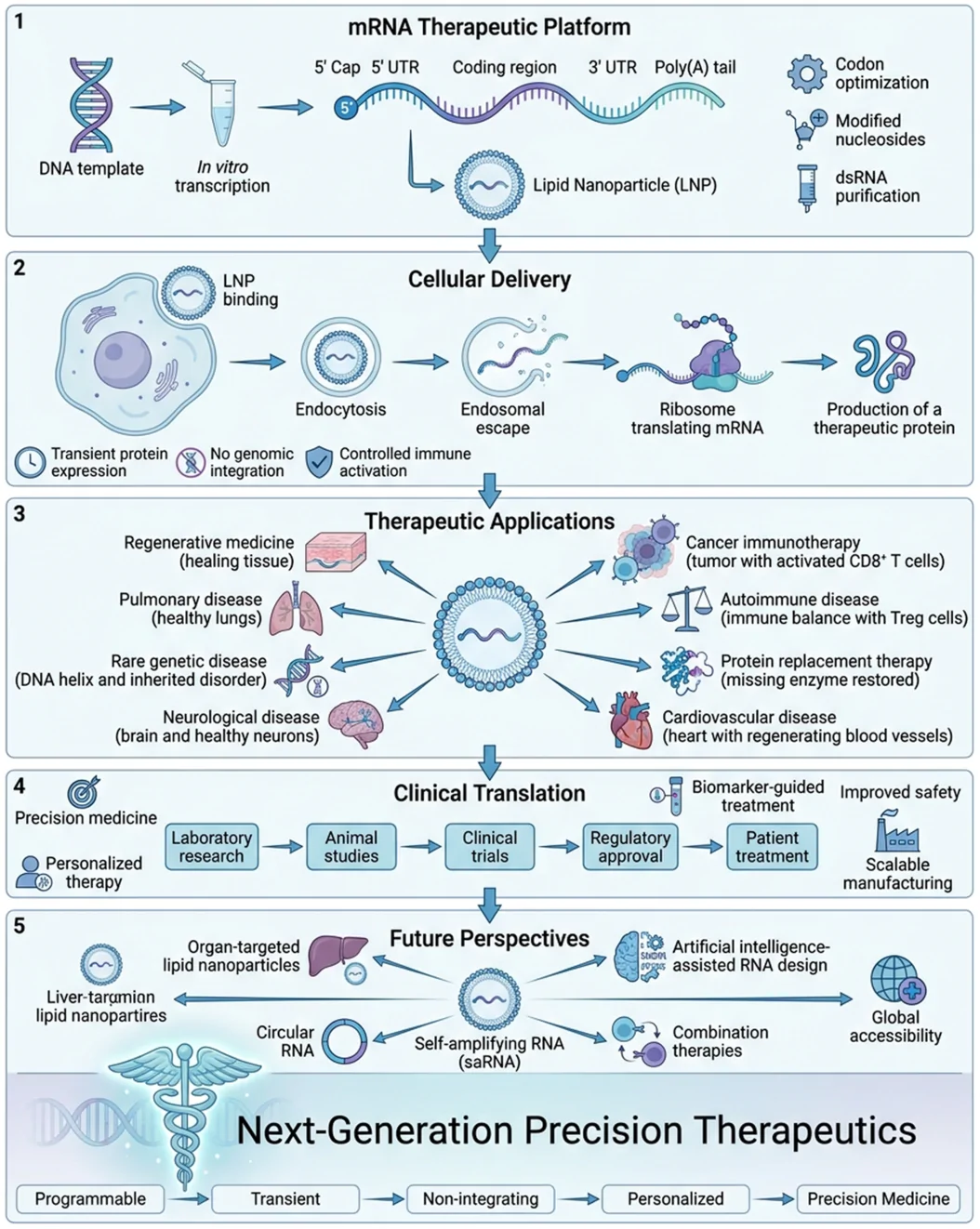

**Abbreviations**
AI: artificial intelligence
APC: antigen‐presenting cell
BBB: blood–brain barrier
CD8^+^
: cluster of differentiation 8 positive T cell
CNS: central nervous system
COVID‐19: coronavirus disease 2019
CTLA‐4: cytotoxic T‐lymphocyte‐associated protein 4
dsRNA: double‐stranded RNA
FDA: Food and Drug Administration
GMP: good manufacturing practice
HLA: human leukocyte antigen
IRES: internal ribosome entry site
LNP: lipid nanoparticle
MHC: major histocompatibility complex
mRNA: messenger ribonucleic acid
NSCLC: non‐small cell lung cancer
ORF: open reading frame
PD‐1: programmed cell death protein 1
PD‐L1: programmed death‐ligand 1
PEG: polyethylene glycol
PK/PD: pharmacokinetics/pharmacodynamics
PRR: pattern recognition receptor
RNA: ribonucleic acid
saRNA: self‐amplifying RNA
TAA: tumor‐associated antigen
Treg: regulatory T cell
UTR: untranslated region
VEGF: vascular endothelial growth factor

## 1 Introduction

Messenger RNA (mRNA) vaccine technology has emerged as a transformative platform in modern biomedicine, offering a flexible and rapidly adaptable approach to disease prevention and treatment [1, 2]. Unlike traditional vaccines that rely on attenuated pathogens or protein subunits, mRNA vaccines utilize synthetic mRNA molecules encoding specific antigens, enabling host cells to transiently produce the target protein and elicit immune responses. This platform is inherently versatile, allowing rapid design, scalable manufacturing, and precise antigen targeting, positioning it as a promising tool not only for infectious diseases but also for a broad range of therapeutic applications [3, 4, 5].

Despite its conceptual origins dating back several decades, the clinical translation of mRNA‐based therapeutics was historically hindered by multiple technical challenges. Early limitations included the inherent instability of mRNA molecules, inefficient in vivo delivery, and unintended activation of innate immune responses leading to reduced protein expression and increased reactogenicity. Over the past decade, however, significant breakthroughs have addressed these barriers [6]. Advances in nucleoside modification, such as the incorporation of modified bases to reduce immunogenicity, alongside the development of lipid nanoparticle (LNP) delivery systems, have dramatically improved mRNA stability, cellular uptake, and translational efficiency. These innovations have collectively enabled the successful deployment of mRNA platforms in clinical settings [7].

The global success of mRNA vaccines during the COVID‐19 pandemic marked a pivotal turning point in the field. Vaccines developed by companies such as Moderna and BioNTech demonstrated unprecedented speed in development, high efficacy, and acceptable safety profiles in large‐scale clinical trials conducted from 2020 onward. This rapid validation not only established mRNA as a viable vaccine modality but also accelerated research and investment into next‐generation mRNA technologies. Consequently, the pandemic catalyzed a paradigm shift, transforming mRNA from an experimental concept into a clinically validated platform with broad therapeutic potential [8, 9].

Building on this momentum, there is growing interest in extending mRNA‐based approaches beyond infectious disease prevention into diverse areas of medicine. The ability of mRNA to encode virtually any protein has opened new avenues in cancer immunotherapy, where personalized vaccines targeting tumor‐specific neoantigens are being actively investigated [2]. Similarly, mRNA therapeutics are being explored for protein replacement in genetic disorders, modulation of immune responses in autoimmune diseases, and regeneration of damaged tissues in cardiovascular and neurological conditions [10]. These emerging applications highlight the unique capacity of mRNA platforms to bridge vaccination and therapeutic intervention, effectively blurring the traditional boundaries between prophylaxis and treatment.

The expansion of mRNA technology into non‐infectious disease domains is driven by several key advantages, including its transient expression profile, lack of genomic integration, and adaptability to personalized medicine approaches. However, challenges remain, particularly in achieving targeted delivery to specific tissues, minimizing adverse immune reactions, and ensuring long‐term safety and cost‐effective production. Addressing these issues will be critical for the successful clinical translation of mRNA therapeutics across diverse indications [4].

In this review, I provide a comprehensive overview of mRNA technology, with particular emphasis on its expanding therapeutic applications beyond infectious diseases. I discuss the fundamental principles underlying mRNA design and delivery, evaluate current and emerging clinical applications, and highlight recent technological advancements shaping the future of the field. Furthermore, I examine the key challenges and opportunities that will influence the integration of mRNA‐based therapeutics into mainstream clinical practice. By synthesizing key developments from the past 5 years, this review aims to offer a timely and forward‐looking perspective on the evolving role of mRNA in modern medicine.

## 2 Fundamentals of mRNA Vaccine Technology

### 2.1 Structure and Design of mRNA Constructs

**Figure 1:**
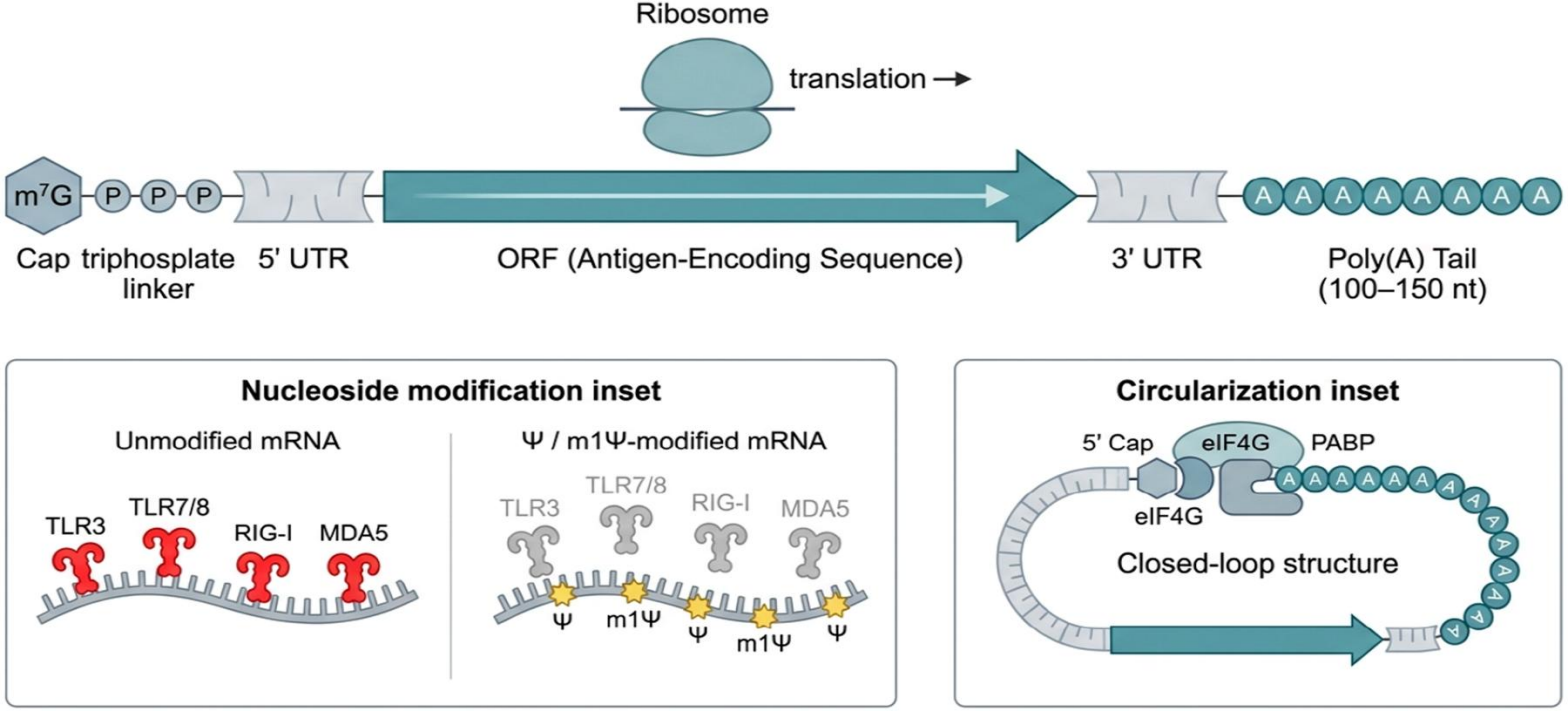
Structural design and optimization of mRNA constructs for vaccine applications. A schematic representation of a typical mRNA construct highlighting key structural elements, including the 5′ cap (m7G), untranslated regions (UTRs), open reading frame (ORF), and poly(A) tail, each contributing to mRNA stability and translational efficiency. The 5′ cap facilitates ribosome recruitment and protects against exonuclease degradation, while optimized 5′ UTR sequences enhance translation initiation. The ORF encodes the antigen of interest and is often optimized through codon usage and sequence engineering. The 3′ UTR regulates transcript stability via interactions with RNA‐binding proteins, and the poly(A) tail promotes mRNA circularization and sustained protein expression. Incorporation of modified nucleosides, such as pseudouridine and N1‐methyl‐pseudouridine, reduces activation of innate immune sensors (e.g., Toll‐like receptors and RIG‐I‐like receptors) while enhancing translational efficiency. Copyright © 2026 Gedion Mengistu Dejen. This figure is an original work created by the author.

The efficacy of mRNA vaccines is fundamentally governed by the rational engineering of the mRNA molecule, which is designed to emulate endogenous eukaryotic messenger RNA while incorporating strategic modifications to enhance stability, translational efficiency, and immunological performance. A canonical mRNA construct comprises a 5′ cap structure (m7G cap), 5′ untranslated region (UTR), open reading frame (ORF), 3′ UTR, and a poly(A) tail (Figure 1), each contributing in a coordinated manner to the regulation of mRNA fate and protein expression [11].

The 5′ cap structure, typically a 7‐methylguanosine (m7G) linked via a 5′–5′ triphosphate bridge, plays a pivotal role in protecting mRNA from exonucleolytic degradation and facilitating cap‐dependent translation initiation through recruitment of eukaryotic initiation factors (eIFs). Recent advances have introduced optimized cap analogs, such as anti‐reverse cap analogs (ARCAs) and CleanCap technologies, which further enhance translational efficiency and ensure correct cap orientation, thereby improving protein yield [12, 13].

The 5′ and 3′ untranslated regions (UTRs) serve as critical regulatory elements that modulate mRNA stability, localization, and translational kinetics. The 5′ UTR influences ribosome scanning and initiation efficiency, often optimized to minimize secondary structures that impede translation. Conversely, the 3′ UTR interacts with RNA‐binding proteins and microRNAs, contributing to transcript stability and longevity. Selection or engineering of UTR sequences derived from highly expressed endogenous genes (e.g., α‐ or β‐globin) has been shown to significantly enhance protein expression in mRNA vaccine platforms [14].

The open reading frame (ORF) encodes the antigen of interest and is typically optimized through codon usage adaptation to match host tRNA abundance, thereby improving translational efficiency. Additional refinements include modulation of GC content and removal of cryptic splice sites or destabilizing motifs. In the context of vaccines, ORF design may also incorporate structural stabilizing mutations in encoded proteins (e.g., prefusion stabilization of viral glycoproteins) to maximize immunogenicity and antigen fidelity.

The poly(A) tail, usually consisting of 100–150 adenine residues, enhances mRNA stability and translation by promoting circularization of the mRNA through interactions with poly(A)‐binding proteins (PABPs) and the translation initiation complex. This closed‐loop structure facilitates ribosome recycling and sustained protein production. Both enzymatic polyadenylation and template‐encoded poly(A) tails are employed, with the latter offering greater control over tail length and consistency [15].

A transformative advancement in mRNA technology has been the incorporation of modified nucleosides, particularly pseudouridine (Ψ) and N1‐methyl‐pseudouridine (m1Ψ). These modifications reduce activation of innate immune sensors such as Toll‐like receptors (TLR3, TLR7, TLR8) and cytosolic receptors like RIG‐I and MDA5, thereby preventing excessive interferon responses that can inhibit translation. At the same time, nucleoside modification enhances ribosomal decoding efficiency and increases protein output. This dual benefit, attenuation of innate immunogenicity coupled with enhanced translational capacity, has been central to the clinical success of mRNA vaccines developed during and after the COVID‐19 pandemic [16, 17].

Beyond these core elements, additional design considerations further refine mRNA performance. These include sequence engineering to reduce double‐stranded RNA contaminants, incorporation of optimized untranslated regions to fine‐tune expression kinetics, and purification strategies such as high‐performance liquid chromatography (HPLC) to improve transcript quality [18]. Collectively, these innovations underscore the importance of integrated mRNA design, where structural elements and chemical modifications are synergistically optimized to achieve robust, safe, and durable protein expression.

### 2.2 Delivery Systems

**Figure 2:**
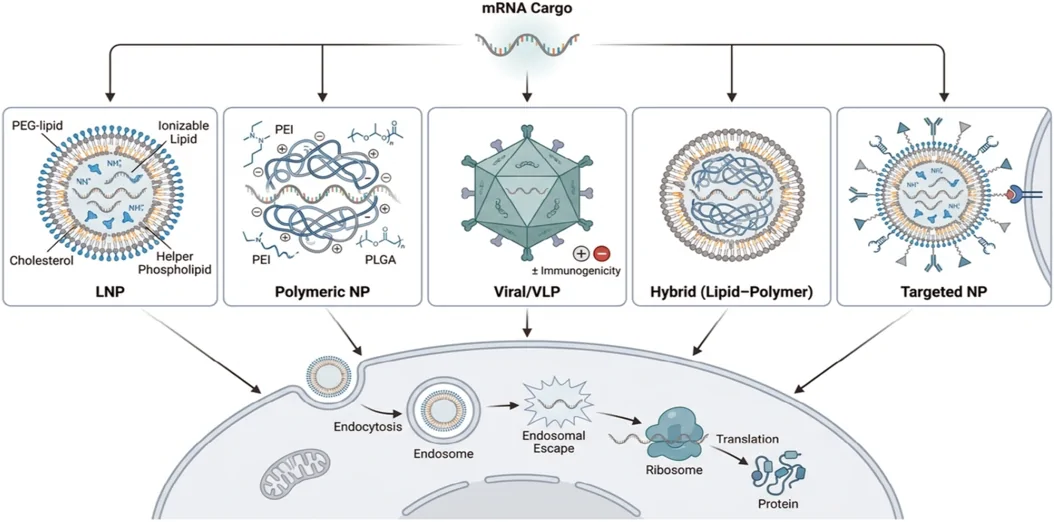
mRNA delivery systems: platforms and mechanisms. Schematic representation of major mRNA delivery platforms and their mechanisms of action. Following administration, mRNA‐loaded nanoparticles are internalized by cells via endocytosis, undergo endosomal escape, and release mRNA into the cytoplasm for translation into protein, leading to antigen expression or therapeutic effects. Lipid nanoparticles (LNPs) enable efficient encapsulation, protection, and cytosolic delivery. Polymeric systems provide tunable properties and controlled release but may have lower efficiency. Viral and virus‐like systems offer high delivery efficiency with potential immunogenicity concerns. Hybrid platforms combine lipid and polymer advantages, while targeted systems enhance cell‐specific delivery and precision. Common challenges include stability, delivery efficiency, safety, and scalability. Copyright © 2026 Gedion Mengistu Dejen. This figure is an original work created by the author.

Efficient intracellular delivery of mRNA remains a central determinant of the efficacy and safety of mRNA‐based vaccines and therapeutics. In its naked form, mRNA is inherently unstable due to its large molecular size, polyanionic nature, and rapid degradation by ubiquitous extracellular ribonucleases (Figure 2). Moreover, the negatively charged phosphate backbone of mRNA prevents passive diffusion across the hydrophobic lipid bilayer of cellular membranes. These intrinsic barriers necessitate the use of sophisticated delivery systems capable of protecting mRNA, facilitating cellular uptake, and ensuring cytosolic release where translation occurs [19, 20].

#### 2.2.1 Lipid Nanoparticles (LNPs)

Among available delivery platforms, lipid nanoparticles (LNPs) have emerged as the most clinically advanced and widely adopted system, underpinning the success of mRNA vaccines developed during the COVID‐19 pandemic. LNPs are typically composed of four key components: ionizable lipids, helper phospholipids, cholesterol, and polyethylene glycol (PEG)‐lipids, each fulfilling distinct structural and functional roles [21, 22].

Ionizable lipids are the core component, designed to remain neutrally charged at physiological pH but become protonated under acidic conditions, such as within endosomes. This pH‐responsive behavior facilitates endosomal escape by promoting membrane destabilization and fusion, thereby enabling the release of mRNA into the cytoplasm. Helper lipids, including phospholipids and cholesterol, contribute to nanoparticle stability, membrane fluidity, and structural integrity, while PEG‐lipids improve colloidal stability, reduce aggregation, and prolong systemic circulation time [23].

Following administration, LNPs are internalized by cells primarily through endocytosis, particularly by antigen‐presenting cells such as dendritic cells. Once inside the endosomal compartment, the protonation of ionizable lipids induces disruption of the endosomal membrane, allowing mRNA to escape into the cytosol for translation. Despite their success, LNPs are associated with certain limitations, including dose‐dependent reactogenicity, potential complement activation, and limited tissue specificity, which have prompted ongoing efforts to refine their composition and targeting capabilities [24].

#### 2.2.2 Polymeric Nanoparticles

Polymeric delivery systems represent a versatile alternative to lipid‐based carriers, offering tunable physicochemical properties and controlled release kinetics. Polymers such as polyethylenimine (PEI) and poly (lactic‐co‐glycolic acid) (PLGA) can form electrostatic complexes with mRNA, protecting it from enzymatic degradation and facilitating cellular uptake. These systems can be engineered to optimize particle size, charge density, and biodegradability, enabling customization for specific therapeutic applications [25].

However, polymeric carriers often face challenges related to cytotoxicity and lower transfection efficiency compared to LNPs, particularly with highly cationic polymers like PEI. Recent innovations focus on developing biodegradable and less toxic polymer formulations, as well as hybrid lipid–polymer systems that combine the advantages of both platforms [26].

#### 2.2.3 Viral and Hybrid Delivery Systems

Viral vectors, including adenoviral and lentiviral systems, have long been utilized for nucleic acid delivery due to their high transduction efficiency. While they are less commonly used for mRNA vaccines compared to DNA‐based approaches, viral and virus‐like particle (VLP) systems are being explored to enhance intracellular delivery of RNA [27]. These systems can achieve efficient cellular entry and expression but are often limited by immunogenicity, pre‐existing immunity, and regulatory complexities, which restrict their widespread application.

To address these limitations, hybrid delivery systems that combine viral and non‐viral elements are under development. These platforms aim to retain the high efficiency of viral systems while improving safety and manufacturability, representing a promising direction for next‐generation mRNA delivery technologies [28].

#### 2.2.4 Emerging Strategies for Targeted Delivery

A major frontier in mRNA delivery is the development of targeted delivery systems that enable selective uptake by specific cell types or tissues. Strategies include surface modification of nanoparticles with ligands, antibodies, or aptamers that recognize cell‐specific receptors, thereby enhancing delivery to key immune cells such as dendritic cells or to diseased tissues [29].

Additionally, advances in organ‐specific LNP formulations have demonstrated preferential delivery to organs such as the liver, spleen, or lungs, depending on lipid composition and physicochemical properties. This has significant implications for expanding mRNA therapeutics beyond vaccines into areas such as cancer immunotherapy, protein replacement therapy, and regenerative medicine [30].

Collectively, the evolution of mRNA delivery systems, from conventional lipid nanoparticles to advanced targeted and hybrid platforms, has been instrumental in overcoming the intrinsic barriers associated with mRNA therapeutics. Continued innovation in delivery technologies will be essential for improving tissue specificity, reducing adverse effects, and unlocking the full therapeutic potential of mRNA across a broad spectrum of diseases.

### 2.3 Mechanisms of Action

**Figure 3:**
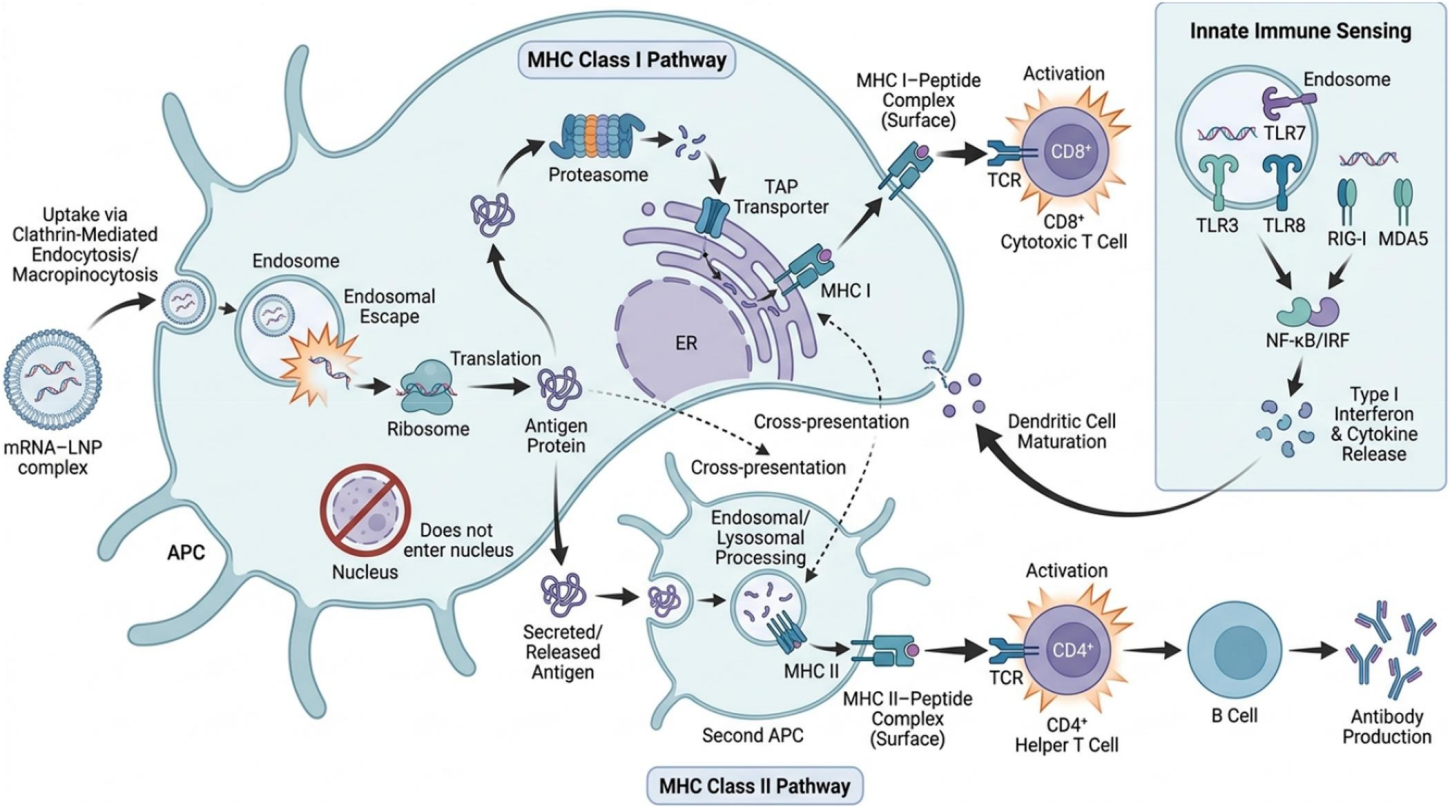
Mechanism of action of mRNA vaccines. mRNA–lipid nanoparticle complexes are internalized by antigen‐presenting cells via endocytosis and release mRNA into the cytoplasm following endosomal escape. The mRNA is translated into antigenic protein, which is processed and presented via MHC class I and II pathways, leading to activation of CD8^+^ cytotoxic T cells and CD4^+^ helper T cells, respectively. Subsequent B cell activation drives antibody production, while innate immune sensing enhances overall immune responses. Copyright © 2026 Gedion Mengistu Dejen. This figure is an original work created by the author.

The mechanism of action of mRNA vaccines is a highly coordinated, multistep process that integrates intracellular delivery, antigen expression, and immune system activation to generate robust and antigen‐specific responses (Figure 3). This process bridges innate and adaptive immunity, ultimately leading to both humoral and cellular immune protection [31].

Following administration, mRNA formulated within lipid nanoparticles (mRNA–LNP complexes) is delivered to target tissues, where it is preferentially taken up by antigen‐presenting cells (APCs), including dendritic cells and macrophages, through endocytic pathways such as clathrin‐mediated endocytosis and macropinocytosis. Once internalized, the nanoparticles are trafficked into endosomal compartments. A critical step in this process is endosomal escape, during which the lipid components destabilize the endosomal membrane, enabling the release of mRNA into the cytoplasm [32, 33].

In the cytosol, the mRNA is recognized by the host translational machinery and undergoes cap‐dependent translation by ribosomes to produce the encoded antigenic protein. The efficiency and duration of antigen expression depend on mRNA stability, sequence optimization, and intracellular conditions. Importantly, mRNA does not enter the nucleus and does not integrate into the host genome, ensuring a transient and controlled expression profile [34].

The synthesized antigen is subsequently processed through distinct intracellular pathways that facilitate presentation to the immune system. Endogenously produced antigens are degraded by the proteasome into peptide fragments, which are transported into the endoplasmic reticulum via transporter associated with antigen processing (TAP) proteins and loaded onto major histocompatibility complex class I (MHC I) molecules. These peptide–MHC I complex are then presented on the cell surface, leading to the activation of CD8^+^ cytotoxic T lymphocytes (CTLs). Activated CTLs play a crucial role in identifying and eliminating infected or malignant cells expressing the target antigen [35].

In parallel, a proportion of the synthesized antigen may be secreted or released upon cell turnover and subsequently taken up by professional APCs. These exogenous antigens are processed within endosomal/lysosomal compartments and presented via MHC class II (MHC II) molecules to CD4^+^ helper T cells. Activation of CD4^+^ T cells is essential for coordinating immune responses, including the stimulation of B cells to produce antigen‐specific antibodies and the support of cytotoxic T cell responses. Additionally, cross‐presentation mechanisms enable certain APCs to present exogenous antigens on MHC I molecules, further amplifying CD8^+^ T cell activation [36].

Beyond adaptive immunity, mRNA vaccines inherently stimulate the innate immune system, which functions as a built‐in adjuvant. The introduced mRNA and its delivery components are recognized by pattern recognition receptors (PRRs), including endosomal Toll‐like receptors (TLR3, TLR7, and TLR8) and cytosolic RNA sensors such as RIG‐I and MDA5 [31]. Activation of these receptors triggers downstream signaling cascades involving transcription factors such as NF‐κB and IRFs, leading to the production of type I interferons and pro‐inflammatory cytokines. These innate signals enhance antigen presentation, promote dendritic cell maturation, and shape the magnitude and quality of adaptive immune responses [37].

However, the activation of innate immunity must be tightly regulated. Excessive stimulation of PRRs can lead to overproduction of interferons, which may inhibit mRNA translation and reduce antigen expression. Therefore, optimized mRNA design, including nucleoside modifications and purification strategies, is essential to achieve a balanced immune response that maximizes immunogenicity while minimizing detrimental inflammatory effects [38].

Generally, the mechanism of action of mRNA vaccines represents a finely tuned interplay between delivery, antigen expression, and immune activation. This integrated process enables the induction of both cell‐mediated and humoral immunity, providing a strong foundation for their application not only in infectious diseases but also in cancer immunotherapy and other therapeutic areas.

### 2.4 Types of mRNA Platforms

mRNA vaccine technologies can be broadly categorized into three principal platform types, conventional (non‐replicating) mRNA, self‐amplifying RNA (saRNA), and circular RNA (circRNA) [39], each defined by distinct structural features, intracellular behavior, and translational dynamics (Table 1). These platforms have been developed to optimize antigen expression, improve stability, and tailor immune responses for diverse prophylactic and therapeutic applications [52].

**Table 1:** Comprehensive comparison of mRNA platform technologies.

Parameter	Conventional (non‐replicating) mRNA	Self‐Amplifying RNA (saRNA)	Circular RNA (circRNA)	References
Molecular Structure	Linear, capped mRNA with UTRs and poly(A) tail	Linear RNA with replicase genes + subgenomic promoter	Covalently closed circular RNA lacking free ends	[40, 41]
Typical Length	~1–5 kb	~9–12 kb	~1–5 kb (circularized)	[42]
Replication Capability	None	Self‐replicating via RNA‐dependent RNA polymerase	None	[43]
Translation Initiation	Cap‐dependent	Cap‐dependent (subgenomic RNA)	Cap‐independent (IRES/m6A‐mediated)	[44, 45]
Expression Kinetics	Rapid, transient	Delayed but prolonged and amplified	Sustained and prolonged	[46, 47]
Protein Yield	Moderate	High	Moderate to high	[35]
Dose Requirement	Higher	Lower (dose‐sparing)	Potentially lower	[31]
Stability	Moderate	Moderate	High (exonuclease‐resistant)	[48]
Innate Immune Activation	Moderate (modifiable)	Higher (replication intermediates)	Lower to moderate	[18]
Delivery Compatibility	Highly compatible with LNPs	Challenging due to size	Emerging compatibility	[36]
Manufacturing Complexity	Established and scalable	More complex	Developing	[17]
Clinical Maturity	Fully validated	Early–mid clinical stage	Preclinical/early‐stage	[13, 15]
Safety Profile	Well established	Under evaluation	Limited data	[35]
Key Advantages	Simplicity, safety, rapid production	High expression at low dose	High stability, prolonged expression	[49]
Key Limitations	Short‐lived expression, higher dose	Large size, reactogenicity risk	Limited data, translation challenges	[50]
Primary Applications	Vaccines, cancer immunotherapy	Vaccines, pandemic preparedness	Protein replacement, emerging therapies	[51]

*Note:* This table summarizes key structural, functional, and translational differences among major mRNA platform technologies. A dedicated “References” column is included to allow the author to insert specific citations corresponding to each parameter based on journal formatting requirements. Conventional mRNA is the most clinically established platform, while self‐amplifying RNA (saRNA) and circular RNA (circRNA) represent next‐generation systems with enhanced expression efficiency and stability, respectively.

#### 2.4.1 Conventional (Non‐Replicating) mRNA

Conventional mRNA represents the most established and clinically validated platform. It consists of a linear, non‐replicating RNA molecule encoding the antigen of interest, flanked by optimized untranslated regions, a 5′ cap, and a poly(A) tail. Following cytoplasmic delivery, the mRNA is directly translated by host ribosomes, resulting in rapid but transient protein expression [39].

The major advantages of this platform include its structural simplicity, well‐characterized safety profile, and predictable pharmacokinetics. Because it lacks replication machinery, antigen expression is tightly controlled and self‐limiting, reducing the risk of prolonged or excessive immune stimulation. This format underpinned the rapid development and deployment of vaccines during the COVID‐19 pandemic. However, its transient nature often necessitates higher doses or repeated administration to achieve and maintain robust immune responses, particularly in therapeutic settings [53].

#### 2.4.2 Self‐Amplifying RNA (saRNA)

Self‐amplifying RNA (saRNA) is an advanced platform derived from positive‐sense RNA viruses, most commonly alphaviruses. In addition to the antigen‐encoding sequence, saRNA includes genes encoding a replicase complex (RNA‐dependent RNA polymerase), enabling intracellular RNA replication [52].

Once inside the cytoplasm, saRNA initiates a replication cycle that generates multiple copies of subgenomic RNA transcripts encoding the target antigen. This leads to prolonged and amplified antigen expression, often at significantly lower doses compared to conventional mRNA. As a result, saRNA is particularly attractive for dose‐sparing strategies, rapid scalability, and cost‐effective vaccine production [50].

Despite these advantages, saRNA platforms present several challenges. Their larger molecular size can complicate delivery and reduce encapsulation efficiency in lipid nanoparticles. Additionally, the formation of replication intermediates may increase innate immune activation, which, if not properly controlled, can suppress translation or increase reactogenicity. Ongoing optimization efforts focus on balancing replication efficiency with tolerability [51].

#### 2.4.3 Circular RNA (circRNA)

Circular RNA (circRNA) is an emerging next‐generation platform distinguished by its covalently closed‐loop structure, which lacks both 5′ and 3′ ends. This unique topology confers exceptional resistance to exonuclease‐mediated degradation, resulting in enhanced molecular stability and prolonged intracellular persistence [54].

Unlike linear mRNA, circRNA typically relies on cap‐independent translation mechanisms, such as internal ribosome entry sites (IRES) or N6‐methyladenosine (m6A)‐mediated initiation, to recruit ribosomes. This enables sustained protein expression over extended periods, which may be advantageous for applications requiring long‐term therapeutic protein production [55].

Although circRNA holds significant promise due to its stability and durability, it remains in the early stages of development. Key challenges include optimizing translation efficiency, ensuring efficient delivery, and establishing scalable manufacturing processes. Nevertheless, circRNA is increasingly recognized as a potential platform for next‐generation vaccines, protein replacement therapies, and regenerative medicine applications [56].

## 3 Advantages and Limitations of mRNA Therapeutics

Messenger RNA therapeutics have emerged as a transformative modality in modern medicine, offering a unique combination of programmability, manufacturing speed, and broad clinical applicability. Unlike conventional small molecules, recombinant proteins, or DNA‐based gene therapies, mRNA platforms function through transient intracellular delivery of genetic instructions that direct host cells to synthesize therapeutic proteins in situ. This feature enables rapid adaptation to evolving clinical needs while minimizing risks associated with permanent genomic modification. The successful deployment of mRNA vaccines during the COVID‐19 pandemic accelerated global confidence in RNA‐based technologies and highlighted their potential far beyond infectious disease prevention.

Despite these advantages, several scientific and translational barriers continue to limit the full realization of mRNA therapeutics. Challenges related to molecular stability, cold‐chain logistics, innate immune activation, and tissue‐specific delivery remain central to the next phase of platform optimization. A balanced understanding of these strengths and limitations is therefore essential for guiding future innovation and clinical integration [7, 57, 58].

### 3.1 Advantages of mRNA Therapeutics

#### 3.1.1 Rapid Design and Scalable Manufacturing

One of the most compelling advantages of mRNA therapeutics is the speed with which candidate products can be designed, produced, and iteratively optimized. Once the nucleotide sequence of a target antigen or therapeutic protein is known, an mRNA construct can be computationally designed within days through sequence engineering strategies such as codon optimization, untranslated region selection, and nucleoside modification [1]. In contrast to traditional vaccine development, which often requires cell culture adaptation, protein purification, or pathogen propagation, mRNA production relies primarily on cell‐free in vitro transcription processes that are highly modular and readily standardized [59].

This platform‐based manufacturing model allows multiple products to be produced using similar upstream and downstream workflows, reducing development timelines and facilitating rapid scale‐up during public health emergencies. The same manufacturing infrastructure can often be repurposed for different targets by simply changing the encoded sequence. Such agility is particularly valuable for emerging infectious diseases, seasonal vaccine updates, personalized cancer vaccines, and rare disease therapeutics requiring rapid customization [60].

In addition, synthetic manufacturing reduces dependence on living biological systems, thereby minimizing contamination risks, simplifying quality control, and enabling decentralized production strategies. As continuous manufacturing and automated formulation technologies mature, the scalability and cost‐efficiency of mRNA production are expected to improve further [61].

#### 3.1.2 Non‐Integrating and Transient Expression

A defining safety advantage of mRNA therapeutics is that mRNA functions in the cytoplasm and does not require entry into the nucleus, thereby avoiding the need for genomic integration. Unlike certain DNA vectors or integrating viral systems, mRNA does not alter host chromosomal DNA, substantially reducing concerns regarding insertional mutagenesis or permanent off‐target genetic changes [2].

Furthermore, mRNA‐mediated protein expression is inherently transient, as transcripts are naturally degraded through endogenous cellular pathways after translation. This temporary expression profile can be highly advantageous when only short‐term protein production is required, such as vaccination, immune stimulation, regenerative signaling, or episodic protein replacement. It also allows dose titration and repeat administration based on therapeutic need [62].

The transient nature of mRNA may enhance clinical controllability by limiting prolonged exposure to potent biologics and enabling rapid cessation of expression if adverse events occur. This pharmacological reversibility distinguishes mRNA therapeutics from permanent gene‐editing approaches and contributes to their favorable risk–benefit profile in many settings [2].

#### 3.1.3 Versatility Across Diseases and Therapeutic Areas

Another major strength of mRNA technology is its exceptional versatility. Because mRNA can encode virtually any protein, the platform is adaptable across a wide range of medical indications. In prophylactic settings, mRNA vaccines can be rapidly tailored to infectious pathogens. In oncology, personalized mRNA vaccines can encode tumor neoantigens to stimulate targeted antitumor immunity. In genetic disorders, mRNA can transiently replace missing or dysfunctional proteins without permanent genome modification [5].

Additional applications include delivery of cytokines, antibodies, growth factors, enzymes, and genome‐editing proteins such as CRISPR‐associated nucleases. mRNA is also being explored in regenerative medicine for tissue repair, angiogenesis, and stem cell modulation. This breadth of utility is enabled by a common technological backbone in which only the encoded sequence changes while formulation and manufacturing principles remain similar [63].

Importantly, the same platform can support both individualized precision medicine and population‐scale products, making mRNA therapeutics uniquely positioned at the intersection of personalized and global healthcare.

### 3.2 Limitations of mRNA Therapeutics

#### 3.2.1 Stability Constraints and Cold‐Chain Requirements

Despite substantial advances, mRNA remains intrinsically unstable compared with many conventional therapeutics. Its phosphodiester backbone is susceptible to hydrolysis, and RNA is rapidly degraded by ubiquitous ribonucleases present in biological and environmental settings. Although lipid nanoparticle encapsulation and optimized buffer systems significantly improve stability, many mRNA products still require controlled storage conditions [64].

The need for refrigerated or frozen storage has created logistical challenges, particularly in low‐resource settings and geographically remote regions. Cold‐chain dependence increases transportation costs, complicates stock management, and may limit equitable access. Even after thawing or reconstitution, some formulations have restricted shelf lives, requiring careful handling throughout the supply chain [65].

Future solutions include thermostable formulations, lyophilized preparations, improved excipient systems, and next‐generation RNA chemistries that preserve potency at ambient temperatures. Addressing storage limitations will be essential for broader global deployment of mRNA medicines [65, 66].

#### 3.2.2 Immunogenicity and Reactogenicity

While controlled innate immune activation can be beneficial, especially in vaccine applications, excessive immunostimulation remains a major challenge. Exogenous RNA may be recognized by pattern recognition receptors such as Toll‐like receptors and cytosolic RNA sensors, leading to induction of type I interferons and inflammatory cytokines. These responses can suppress translation efficiency, reduce protein yield, and contribute to systemic or local adverse effects [67].

Clinically, reactogenicity may include injection‐site pain, fever, fatigue, myalgia, chills, or transient inflammatory responses. Although generally manageable, such effects can influence patient acceptance, repeat dosing feasibility, and risk–benefit considerations in chronic therapeutic applications [38].

Advances such as nucleoside modification, removal of double‐stranded RNA impurities, optimized lipid composition, and dose refinement have significantly improved tolerability. Nevertheless, immune responses remain context dependent and may vary according to indication, route of administration, and patient‐specific factors [31].

#### 3.2.3 Delivery Efficiency and Tissue Targeting Challenges

Efficient intracellular delivery remains one of the most significant bottlenecks in the field. Because naked mRNA is large, negatively charged, and membrane impermeable, it requires carrier systems capable of protection, cellular uptake, endosomal escape, and cytosolic release. Lipid nanoparticles currently dominate clinical translation, but delivery efficiency is still imperfect, with only a fraction of administered material reaching the desired intracellular destination [67].

Another major limitation is tissue specificity. Many existing nanoparticle systems exhibit preferential biodistribution to organs such as the liver or uptake by phagocytic cells, which may be advantageous in some contexts but suboptimal for others. Achieving selective delivery to tumors, lungs, muscle, central nervous system tissues, or diseased organs remains an active area of research [68].

Repeated administration may also be complicated by anti‐carrier immune responses, complement activation, or cumulative toxicity. To overcome these barriers, next‐generation approaches are focusing on ligand‐targeted nanoparticles, biodegradable polymers, organ‐selective lipid compositions, extracellular vesicles, and hybrid nanocarriers [69].

## 4 Expanding Therapeutic Applications Beyond Infectious Diseases

The clinical success of mRNA vaccines in infectious disease prevention has catalyzed broader interest in messenger RNA as a versatile therapeutic platform across multiple non‐communicable and chronic disease areas (Table 2). Unlike conventional biologics, mRNA therapeutics enable in situ production of functional proteins within host cells, thereby combining the precision of gene‐based medicine with the flexibility of transient, non‐integrating expression. This capability has opened new avenues in oncology, immune modulation, regenerative medicine, inherited metabolic disorders, cardiovascular repair, and neurological disease [61, 90].

**Table 2:** Current and emerging non‐infectious applications of mRNA therapeutics.

Therapeutic area	Disease examples	mRNA cargo/strategy	Mechanism of action	Potential advantages	Clinical development stage	Key challenges	References
Cancer immunotherapy	Melanoma, NSCLC, colorectal cancer, pancreatic cancer	Tumor‐associated antigens (TAAs), neoantigens, cytokines	Activates APCs, induces CD8^+^ T cells, enhances antitumor immunity	Personalized therapy, adaptable, synergistic with checkpoint inhibitors	Clinical trials/advanced clinical stage	Tumor heterogeneity, immune escape, suppressive microenvironment	[70, 71]
Personalized cancer vaccines	Patient‐specific solid tumors	Individualized neoantigen mRNA constructs	Stimulates mutation‐specific T‐cell responses	Precision oncology, reduced off‐target toxicity	Clinical trials	Rapid sequencing, manufacturing timelines, cost	[72, 73]
Autoimmune diseases	Multiple sclerosis, rheumatoid arthritis, lupus	Autoantigen‐encoding tolerogenic mRNA	Promotes immune tolerance, induces Tregs, suppresses autoreactivity	Antigen‐specific therapy without global immunosuppression	Preclinical/early clinical	Identifying dominant autoantigens, durability	[74, 75]
Inflammatory disorders	IBD, psoriasis, chronic inflammation	Anti‐inflammatory cytokines, immune modulators	Reduces inflammatory signaling pathways	Targeted immunomodulation	Preclinical	Controlled dosing, tissue targeting	[76]
Protein replacement therapy	Hemophilia, cystic fibrosis, α1‐antitrypsin deficiency	mRNA encoding missing/defective proteins	Transient intracellular protein synthesis	Avoids genomic integration, repeat dosing possible	Preclinical/clinical trials	Repeat administration, expression duration	[77, 78]
Metabolic genetic disorders	OTC deficiency, methylmalonic acidemia, PKU	Enzyme‐encoding mRNA	Restores metabolic enzyme activity	Rapid platform adaptation, liver targeting possible	Clinical trials/preclinical	Organ‐specific delivery, chronic dosing	[79, 80]
Cardiovascular diseases	Myocardial infarction, ischemic heart disease, PAD	VEGF mRNA, regenerative growth factors	Promotes angiogenesis, tissue repair, cardioprotection	Temporary regenerative signaling	Early clinical/preclinical	Targeted myocardial delivery, transient effect	[81, 82]
Cardiac regeneration	Heart failure, post‐infarction remodeling	Transcription factors, survival proteins	Enhances cardiomyocyte survival and repair	Novel regenerative strategy	Preclinical	Safety, arrhythmia risk, delivery efficiency	[82, 83]
Neurological disorders	Parkinson's disease, Alzheimer's disease, ALS	Neurotrophic factors, anti‐aggregation proteins	Supports neuronal survival, reduces pathology	Programmable CNS therapy	Preclinical	Blood–brain barrier penetration	[84]
Rare genetic diseases	Lysosomal storage diseases, enzyme deficiencies	Functional enzyme/protein mRNA	Restores deficient protein activity	Precision medicine for ultra‐rare diseases	Preclinical/early clinical	Repeated dosing, cost, access	[72, 85]
Pulmonary disorders	Cystic fibrosis, pulmonary hypertension	CFTR mRNA, vasoregulatory proteins	Restores lung protein function	Local inhaled delivery possible	Clinical trials/preclinical	Mucus barrier, airway delivery efficiency	[86, 87]
Regenerative medicine	Wound healing, tissue injury, musculoskeletal repair	Growth factors, stem‐cell modulators	Enhances tissue regeneration and repair	Temporary biologic stimulation	Preclinical	Spatial control, repeated dosing	[88, 89]

*Note:* This table summarizes major current and emerging applications of mRNA therapeutics beyond infectious diseases. Messenger RNA platforms enable transient, non‐integrating intracellular protein production and can be rapidly adapted to diverse therapeutic indications. Development stages vary by indication, with oncology leading clinical translation, while neurological, regenerative, and many rare disease applications remain in earlier stages. A dedicated reference column is provided for insertion of citations according to target journal style.

A major advantage of mRNA technology in these settings is its modularity. By altering only, the encoded sequence, the same manufacturing and delivery framework can be adapted to express tumor antigens, tolerogenic proteins, missing enzymes, growth factors, or neuroprotective molecules. Furthermore, transient expression may reduce long‐term safety concerns associated with permanent genome editing or integrating viral vectors [63]. As delivery systems improve and clinical experience accumulates, mRNA therapeutics are increasingly positioned as a next‐generation treatment modality extending well beyond vaccination.

### 4.1 Cancer Immunotherapy

Cancer immunotherapy has emerged as one of the most promising frontiers for mRNA technology. The ability of mRNA to encode tumor‐specific antigens and stimulate potent cellular immunity makes it particularly attractive for anticancer vaccine development [70].

#### 4.1.1 Tumor‐Associated Antigen vs. Neoantigen Vaccines

Early mRNA cancer vaccines focused on tumor‐associated antigens (TAAs), which are proteins overexpressed or aberrantly expressed in malignant cells but may also be present at lower levels in normal tissues. Examples include shared cancer antigens involved in cell proliferation or differentiation. While TAAs offer broad applicability across patients, immune tolerance and limited specificity may reduce efficacy [91, 92].

In contrast, neoantigens arise from tumor‐specific somatic mutations and are absent from healthy tissues. These mutated peptides are highly attractive immunological targets because they are recognized as non‐self, enabling stronger and more selective T‐cell responses with lower risk of autoimmunity. mRNA technology is especially suited to neoantigen vaccination because multiple individualized targets can be encoded in a single construct [70, 91].

#### 4.1.2 Personalized Cancer Vaccines

One of the most transformative developments in oncology is the rise of personalized mRNA cancer vaccines. Using next‐generation sequencing, patient tumors can be analyzed to identify unique mutational signatures. Bioinformatic algorithms then predict immunogenic neoepitopes, which are rapidly incorporated into custom mRNA vaccine formulations [93].

These individualized vaccines aim to prime cytotoxic T cells against patient‐specific tumor targets, reducing recurrence risk and enhancing tumor control. Personalized mRNA vaccines are particularly relevant in melanoma, lung cancer, colorectal cancer, and other malignancies with high mutational burden [94].

#### 4.1.3 Combination With Checkpoint Inhibitors

mRNA cancer vaccines are increasingly being evaluated in combination with immune checkpoint inhibitors, such as agents targeting PD‐1, PD‐L1, or CTLA‐4 pathways. While vaccines expand tumor‐specific T‐cell populations, checkpoint blockade helps reverse tumor‐induced immune suppression and T‐cell exhaustion [95].

This complementary strategy may enhance response durability and broaden the proportion of patients who benefit from immunotherapy. Combination approaches are considered a key future direction for integrating mRNA vaccines into mainstream oncology practice.

### 4.2 Autoimmune and Inflammatory Diseases

Beyond immune activation, mRNA therapeutics can also be engineered to promote immune tolerance, creating new opportunities in autoimmune and inflammatory disorders.

#### 4.2.1 Induction of Immune Tolerance

Autoimmune diseases arise when the immune system inappropriately targets self‐antigens. In contrast to vaccine strategies that stimulate immunity, tolerogenic mRNA approaches aim to present disease‐relevant autoantigens in a non‐inflammatory context, thereby promoting regulatory immune pathways [74].

This may include induction of regulatory T cells (Tregs), suppression of autoreactive effector T cells, and reprogramming of antigen‐presenting cells toward a tolerogenic phenotype. Such approaches could offer disease‐specific immune control without broad immunosuppression [96].

For example, in multiple sclerosis (MS), mRNA constructs encoding myelin‐related antigens are being investigated to selectively reduce immune attacks against central nervous system myelin. Also in rheumatoid arthritis, mRNA‐based tolerogenic therapies may target synovial autoantigens or inflammatory mediators to reduce joint destruction [97, 98].

More broadly, mRNA therapeutics may be applicable in systemic lupus erythematosus, inflammatory bowel disease, psoriasis, and other immune‐mediated conditions where antigen‐specific tolerance is desirable.

### 4.3 Protein Replacement Therapy

Protein replacement is another highly promising application of mRNA therapeutics, particularly for diseases caused by absent or dysfunctional proteins.

#### 4.3.1 mRNA Encoding Therapeutic Proteins

Instead of repeatedly administering recombinant proteins, mRNA can instruct patient cells to transiently synthesize the therapeutic protein internally. This may improve bioavailability, permit more natural post‐translational modification, and reduce manufacturing complexity for difficult‐to‐produce proteins [61]. The duration of expression can be modulated through dosing intervals, sequence engineering, and delivery systems, allowing controllable treatment strategies.

#### 4.3.2 Applications in Genetic Disorders

Protein replacement via mRNA is especially relevant for monogenic disorders involving loss‐of‐function mutations. Potential applications include enzyme deficiencies, coagulation disorders, metabolic diseases, and inherited pulmonary or hepatic conditions [85].

Examples under investigation include mRNA encoding clotting factors for hemophilia, enzymes for lysosomal storage diseases, and transport proteins for metabolic syndromes. Because mRNA does not permanently modify DNA, repeat dosing strategies may provide a safer alternative to some gene therapy approaches [77].

#### 4.3.3 Managing Immunogenicity of Newly Introduced Therapeutic Proteins

A key safety consideration for protein replacement mRNA therapies is that patients with genetic or metabolic deficiencies have often never been exposed to the missing protein and may lack immune tolerance to it. Once translated, the newly synthesized protein can be processed and presented by antigen‐presenting cells, creating a risk that it will be recognized as foreign and targeted by anti‐drug antibodies or cytotoxic T‐cell responses that neutralize the therapeutic effect or trigger hypersensitivity reactions. This phenomenon is well documented with recombinant enzyme replacement therapies and is an important consideration for mRNA‐based protein replacement strategies as well [99].

Part of the risk arises from the delivery platform itself: exogenous mRNA and its lipid nanoparticle carrier can activate innate pattern‐recognition receptors, and this innate immune activation can act as an unintended adjuvant that amplifies adaptive immune responses against the encoded protein rather than only against the vector. Strategies to blunt this innate signal, including incorporation of modified nucleosides such as pseudouridine, purification to remove double‐stranded RNA contaminants, and optimized untranslated region design, reduce pattern‐recognition receptor engagement and are therefore expected to lower the likelihood of an antibody or cellular response developing against the therapeutic protein itself [100, 101].

Additional approaches under consideration to minimize immune reactions against newly appearing proteins include transient, low, and intermittent dosing regimens that mimic gradual antigen exposure rather than triggering an abrupt immune‐alerting surge, tissue‐restricted expression that limits systemic antigen presentation, and, where feasible, sequence engineering to reduce immunogenic epitopes while preserving protein function. Because immune tolerance to a newly expressed protein cannot be assumed, careful preclinical immunogenicity screening and clinical monitoring for anti‐drug antibodies remain essential components of the development pathway for mRNA‐based protein replacement therapies.

### 4.4 Cardiovascular Diseases

Cardiovascular disease remains a leading global cause of mortality, and mRNA therapeutics are being explored for tissue repair, vascular regeneration, and cardioprotection [102].

#### 4.4.1 Angiogenic Factors

One major strategy involves delivery of mRNA encoding angiogenic growth factors, particularly vascular endothelial growth factor (VEGF), to stimulate neovascularization in ischemic tissues. Localized VEGF mRNA expression may enhance blood vessel formation, improve perfusion, and support recovery following myocardial infarction or peripheral artery disease. Compared with recombinant proteins, mRNA may provide more efficient transient local expression while avoiding prolonged systemic exposure [103].

#### 4.4.2 Cardiac Repair and Regeneration

mRNA is also being investigated for cardiac regeneration, including expression of survival factors, anti‐fibrotic mediators, and developmental regulators that promote cardiomyocyte repair. Temporary delivery of regenerative signals may enhance tissue healing after ischemic injury while limiting long‐term risks associated with constitutive gene expression. These approaches are particularly attractive because the injured myocardium has limited intrinsic regenerative capacity [81].

### 4.5 Neurological Disorders

Neurological diseases represent a challenging but potentially transformative area for mRNA therapeutics.

#### 4.5.1 Blood–Brain Barrier Challenges

The principal barrier is the blood–brain barrier (BBB), which restricts entry of large molecules and nanoparticle systems into the central nervous system. Efficient and safe delivery to neurons, astrocytes, microglia, or spinal tissues remains a major translational challenge. Current strategies include receptor‐mediated transport systems, intrathecal delivery, intranasal administration, and engineered nanoparticles with BBB‐penetrating ligands [104].

#### 4.5.2 Emerging Applications in Neurodegeneration

mRNA therapeutics are being explored for neurodegenerative disorders such as Parkinson's disease, Alzheimer's disease, amyotrophic lateral sclerosis, and spinal muscular atrophy. Potential applications include expression of neurotrophic factors, enzymes, anti‐aggregation proteins, or gene‐editing components. Transient expression may be advantageous in the nervous system, where precise dose control and reversibility are clinically important [84].

### 4.6 Rare Genetic Diseases

Rare diseases collectively affect millions worldwide, many of whom lack effective treatments. Because a large proportion are monogenic, they are strong candidates for mRNA‐based intervention [72].

#### 4.6.1 Enzyme Deficiencies

Many rare disorders result from deficiency of a single enzyme, transporter, or structural protein. mRNA therapeutics can restore temporary protein function by delivering transcripts encoding the missing product. This is particularly relevant for metabolic disorders affecting the liver, muscle, or hematopoietic system [105].

#### 4.6.2 Precision Medicine Approaches

mRNA platforms are uniquely compatible with precision medicine, as constructs can be rapidly customized to individual mutations, protein variants, or patient‐specific needs. This may be especially valuable for ultra‐rare conditions where conventional drug development is economically challenging. Because manufacturing workflows remain largely constant regardless of encoded sequence, mRNA offers a scalable framework for individualized treatment development [106].

## 5 Clinical Development and Translational Progress

The transformation of messenger RNA therapeutics from a largely experimental concept into a clinically validated medical platform represents one of the most consequential translational advances in modern biomedical science. For decades, the therapeutic use of synthetic RNA was constrained by challenges related to molecular instability, inefficient intracellular delivery, rapid enzymatic degradation, and excessive innate immune activation. These limitations initially positioned mRNA as a promising but technically immature modality. However, sustained progress in RNA chemistry, sequence engineering, purification methods, and nanocarrier technologies fundamentally changed this outlook and enabled the transition of mRNA therapeutics into mainstream clinical development [49, 107].

The global deployment of mRNA vaccines during the COVID‐19 pandemic served as a pivotal proof of concept, demonstrating that mRNA products could be designed rapidly, manufactured at unprecedented scale, distributed internationally, and achieve robust real‐world efficacy [49]. This milestone had implications far beyond infectious disease prevention. It validated the broader proposition that transient delivery of genetic instructions could be safely translated into therapeutic proteins within the human body. As a result, investment in mRNA technologies expanded dramatically across oncology, cardiovascular medicine, rare diseases, autoimmune disorders, regenerative medicine, and protein replacement therapy [108].

Clinical translation of mRNA therapeutics now depends on an integrated continuum of innovation that spans preclinical validation, carefully designed human trials, scalable manufacturing systems, and evolving regulatory frameworks. Unlike conventional small molecules, mRNA medicines are hybrid products in which therapeutic performance is determined by both the encoded nucleic acid sequence and the delivery vehicle used to transport it into cells. Consequently, translational progress in this field reflects advances not only in biology, but also in materials science, analytical chemistry, process engineering, quality systems, and regulatory science [109].

As the field enters a post‐pandemic phase of maturation, attention is increasingly shifting from emergency vaccine deployment to durable therapeutic applications requiring repeated dosing, long‐term benefit, organ‐selective delivery, and sophisticated clinical endpoints. This next stage of development will determine whether mRNA therapeutics become a broadly applicable platform across medicine or remain concentrated in select indications [93].

### 5.1 Preclinical Advances

Preclinical research has played a foundational role in establishing the scientific legitimacy and therapeutic potential of mRNA‐based medicines. Before any clinical application became feasible, investigators needed to demonstrate that synthetic mRNA could be introduced into living cells, evade immediate degradation, engage the host translational machinery, and produce biologically functional proteins at therapeutically meaningful levels [110]. These early milestones were essential because they directly addressed skepticism regarding whether exogenous RNA could function efficiently in vivo.

Subsequent advances in nucleoside modification, particularly the incorporation of modified uridines, substantially reduced innate immune recognition and improved translational efficiency. Parallel improvements in chromatographic purification enabled the removal of double‐stranded RNA contaminants generated during in vitro transcription, thereby reducing inflammatory responses that previously limited therapeutic utility. Together, these molecular refinements transformed mRNA from a highly immunostimulatory laboratory reagent into a clinically viable therapeutic substrate [100].

Equally important was the emergence of modern delivery systems, especially lipid nanoparticles, which solved one of the field's most persistent bottlenecks: efficient intracellular delivery. Without protective carriers, naked mRNA is rapidly degraded in extracellular environments and cannot readily cross cell membranes because of its size and negative charge. Nanoparticle technologies enabled protection from ribonucleases, enhanced cellular uptake, endosomal escape, and cytoplasmic release, thereby making systemic and local delivery strategies clinically realistic [97, 104].

#### 5.1.1 Animal Models and Proof‐of‐Concept Studies

Animal studies have provided critical evidence that mRNA therapeutics can generate meaningful biological effects across diverse disease models. In vaccine research, murine and non‐human primate studies consistently demonstrated that mRNA immunization can induce potent neutralizing antibody responses, helper T‐cell activation, and cytotoxic CD8^+^ T‐cell responses. These findings established the immunological foundation later confirmed in large‐scale human vaccine trials [111, 112].

In oncology, preclinical tumor models showed that mRNA vaccines encoding tumor‐associated antigens or personalized neoantigens could stimulate antigen‐specific T‐cell responses capable of delaying tumor growth or improving survival. These effects became even more pronounced when mRNA vaccines were combined with checkpoint inhibitors, highlighting the role of combination immunotherapy in overcoming tumor‐induced immune suppression [71].

In the field of inherited disease, liver‐directed mRNA delivery has been especially influential. Rodent and primate studies demonstrated that mRNA encapsulated in lipid nanoparticles could drive transient hepatic expression of enzymes, coagulation factors, or secreted proteins at levels sufficient to correct biochemical abnormalities [113]. These studies significantly advanced the rationale for treating metabolic and monogenic disorders using repeat‐dose mRNA therapy.

Cardiovascular models have shown that localized delivery of mRNA encoding angiogenic factors such as VEGF can improve tissue perfusion following ischemic injury. Additional studies suggest that transient expression of regenerative or anti‐apoptotic proteins may support myocardial recovery after infarction. In neurological disease, although central nervous system delivery remains difficult, preclinical work has demonstrated proof‐of‐concept for mRNA‐mediated expression of neurotrophic factors and protective proteins when appropriate delivery routes are used [81, 114].

#### 5.1.2 Toxicology, Biodistribution, and Dose Optimization

Modern preclinical programs extend far beyond efficacy demonstration. Toxicology studies are required to characterize local reactogenicity, systemic inflammatory responses, organ toxicity, complement activation, and repeat‐dose tolerability. These evaluations are particularly important for nanoparticle‐based formulations, as biodistribution patterns often determine both therapeutic activity and off‐target risk [99, 110].

Biodistribution studies using imaging and molecular quantification techniques have shown that many current lipid nanoparticle systems preferentially accumulate in organs such as the liver and spleen. While this can be advantageous for hepatic or immune‐targeted therapies, it also underscores the need for more precise delivery systems for lung, cardiac, muscular, or neurological indications [115].

Dose‐ranging studies further reveal that therapeutic success depends on multiple interacting variables, including mRNA sequence design, route of administration, formulation composition, dosing interval, and host immune status. Collectively, preclinical research has substantially de‐risked the transition of mRNA therapeutics into human clinical testing and continues to guide next‐generation platform design.

### 5.2 Clinical Trials Landscape

The clinical trial landscape for mRNA therapeutics has expanded rapidly from a narrow set of experimental vaccine studies into a broad and increasingly sophisticated development ecosystem. Initial trials primarily sought to establish basic safety, tolerability, and immunogenicity. Today, many programs are designed to evaluate efficacy endpoints, biomarker modulation, progression‐free survival, organ function restoration, and combination treatment strategies [72].

The successful authorization and global administration of mRNA vaccines represented a watershed moment because it converted theoretical platform potential into real‐world evidence. This achievement substantially reduced perceived regulatory and commercial risk, leading to accelerated investment in therapeutic pipelines beyond infectious disease.

#### 5.2.1 Ongoing and Completed Trials

Completed vaccine trials demonstrated that mRNA products could achieve high levels of efficacy with acceptable safety profiles across large populations. These outcomes created confidence in the scalability and reproducibility of mRNA manufacturing processes and supported the broader use of RNA platforms in medicine [116].

In oncology, numerous ongoing studies are investigating individualized neoantigen vaccines, shared tumor‐antigen vaccines, and mRNA‐encoded immune stimulators. These trials span melanoma, lung cancer, colorectal cancer, pancreatic cancer, ovarian cancer, and other solid tumors. Many protocols incorporate checkpoint inhibitors to enhance T‐cell persistence and overcome tumor immune evasion [70, 92].

Rare disease and metabolic programs are increasingly focused on systemic protein replacement. Clinical candidates aim to restore missing enzymes, clotting factors, or transport proteins through repeated dosing strategies, particularly using liver‐targeted delivery systems [106]. These studies are especially important because they test whether mRNA can transition from episodic vaccination to chronic therapeutic use.

Emerging clinical programs are also evaluating localized regenerative applications, including intramyocardial or tissue‐directed delivery of reparative proteins [83]. Although still early in development, such studies signal expansion of mRNA medicine into non‐immunological therapeutic domains.

#### 5.2.2 Key Industry Leaders and Translational Drivers

Several biotechnology companies have played central roles in shaping the field. Moderna has developed one of the broadest mRNA pipelines, spanning vaccines, oncology, rare disease therapeutics, and systemic secreted proteins. BioNTech has been particularly influential in personalized cancer vaccines, immuno‐oncology combinations, and infectious disease programs [117, 118].

Other important contributors include CureVac, which pioneered early RNA therapeutics research, and Arcturus Therapeutics, which has advanced self‐amplifying RNA and next‐generation delivery systems. Numerous academic spinouts, contract development organizations, and strategic partnerships have also accelerated innovation [119].

The clinical ecosystem is now characterized by increasing specialization. Some developers focus on personalized oncology manufacturing, others on organ‐targeted nanoparticles, thermostable formulations, rare disease pipelines, or immune modulation platforms [97]. This diversification suggests long‐term industrial maturity rather than a transient pandemic‐driven phenomenon.

#### 5.2.3 Evolving Clinical Trial Design

Modern mRNA trials increasingly incorporate adaptive protocols, biomarker‐driven enrollment, translational immune‐monitoring, and combination therapy frameworks. Personalized cancer vaccine studies often integrate genomic sequencing, neoantigen prioritization, and individualized batch manufacturing under compressed timelines. Chronic disease programs emphasize repeat‐dose safety, durability of expression, and immunogenicity management [94]. These evolving designs reflect a shift from proving that mRNA works to determining where it works best and how it can outperform existing therapeutic modalities.

### 5.3 Regulatory Considerations

The regulatory oversight of mRNA therapeutics is uniquely complex because these products combine characteristics of biologics, nucleic acid medicines, vaccine platforms, and nanotechnology‐enabled drug delivery systems. Regulators must therefore evaluate not only the biological activity of the encoded protein, but also the quality, consistency, biodistribution, and safety of the RNA molecule and its carrier system [120].

Although early emergency authorizations accelerated public familiarity with mRNA products, long‐term therapeutic indications require more conventional evidentiary standards, including chronic toxicology, manufacturing comparability, long‐duration follow‐up, and indication‐specific efficacy data [18].

#### 5.3.1 Safety Evaluation

Safety assessment typically includes evaluation of local and systemic reactogenicity, inflammatory cytokine responses, hypersensitivity reactions, organ toxicity, and repeat‐dose tolerability. Because mRNA does not integrate into the genome and is naturally degraded over time, it benefits from an intrinsically favorable safety concept compared with permanent gene‐modifying systems. However, transient expression alone does not eliminate the need for careful risk assessment [121].

For repeated systemic therapies, regulators may require detailed monitoring for anti‐carrier immune responses, complement activation, altered pharmacokinetics over time, and cumulative toxicity. In immuno‐oncology settings, exaggerated immune activation or autoimmune phenomena may also require surveillance. Reproductive and developmental toxicology may be necessary depending on the target population and indication [122].

#### 5.3.2 Manufacturing and Quality Control

Manufacturing consistency is a central regulatory priority because relatively small process variations can alter potency, tolerability, or stability. Quality review generally encompasses template DNA integrity, transcription fidelity, RNA purity, residual enzyme or DNA contamination, and levels of double‐stranded RNA impurities [123].

For nanoparticle formulations, regulators typically examine particle size distribution, polydispersity, encapsulation efficiency, surface characteristics, sterility, endotoxin control, and storage stability. Batch‐to‐batch reproducibility is essential, particularly for products manufactured at global scale or across multiple facilities [98].

Comparability studies are especially important when processes change during scale‐up, technology transfer, or formulation refinement. Because mRNA platforms evolve rapidly, regulatory systems must balance innovation with rigorous product consistency standards.

#### 5.3.3 Future Regulatory Directions

As the field diversifies, regulatory science will need to adapt to individualized manufacturing, rapid sequence updates, decentralized production models, and novel carriers such as polymeric nanoparticles, hybrid systems, or extracellular vesicles. Platform‐based regulatory pathways may become increasingly valuable, allowing previously validated manufacturing and delivery frameworks to support faster review of sequence‐modified products [124].

International harmonization of analytical standards, pharmacovigilance expectations, and quality benchmarks will be critical for global expansion. The long‐term success of mRNA therapeutics will depend not only on scientific innovation, but also on efficient, transparent, and scientifically robust regulatory pathways.

## 6 Technological Innovations Driving the Field

The rapid expansion of messenger RNA therapeutics has been enabled not only by successful clinical proof‐of‐concept studies, but also by continuous technological innovation across multiple disciplines. Advances in nanomedicine, synthetic biology, computational sequence design, manufacturing science, and translational pharmacology have collectively transformed mRNA from a fragile laboratory molecule into a programmable therapeutic platform with expanding clinical utility. While first‐generation mRNA products demonstrated the feasibility of transient intracellular protein expression, next‐generation technologies are now focused on improving precision, durability, tolerability, and disease‐specific performance [107].

Three innovation domains are particularly influential in shaping the future of the field. First, delivery systems are evolving from broadly biodistributed carriers toward tissue‐targeted and organ‐selective platforms capable of directing mRNA to specific cell populations. Second, mRNA engineering strategies are enhancing translational efficiency while minimizing innate immune recognition and instability. Third, combination approaches are integrating mRNA therapeutics with established treatment modalities, thereby leveraging complementary mechanisms of action [125].

Together, these advances are shifting mRNA medicine from a generalized platform technology toward a highly optimized and indication‐specific therapeutic modality.

### 6.1 Next‐Generation Delivery Systems

Efficient intracellular delivery remains one of the most decisive determinants of mRNA therapeutic success. Because naked mRNA is large, negatively charged, and highly susceptible to extracellular degradation, clinically meaningful activity depends on carrier systems that can protect the transcript, facilitate cellular uptake, promote endosomal escape, and release cargo into the cytoplasm [126]. First‐generation lipid nanoparticle systems successfully solved many of these challenges and enabled the initial wave of approved mRNA products. However, broader therapeutic applications require a new generation of delivery technologies with greater precision and expanded tissue reach [24].

#### 6.1.1 Targeted Lipid Nanoparticles

Conventional lipid nanoparticles frequently exhibit passive biodistribution patterns dominated by uptake in the liver, spleen, and cells of the reticuloendothelial system. While advantageous for hepatic protein replacement or vaccine applications, these patterns are suboptimal for diseases requiring selective delivery to tumors, lungs, muscle, heart, or immune cell subsets. As a result, substantial effort is now directed toward targeted lipid nanoparticles engineered to recognize specific tissues or cell surface markers [127].

Targeting strategies include incorporation of ligands such as peptides, antibodies, aptamers, sugars, or receptor‐binding molecules onto nanoparticle surfaces. These modifications can enhance uptake by selected cell populations, including dendritic cells, hepatocyte subtypes, tumor cells, endothelial cells, or inflamed tissues. In oncology, targeted nanoparticles may improve intratumoral accumulation while reducing systemic exposure [128]. In immunology, selective delivery to antigen‐presenting cells can increase potency at lower doses.

Another important innovation is the rational redesign of ionizable lipids to optimize pKa, membrane fusion behavior, biodegradability, and intracellular trafficking. Improved lipid chemistry can simultaneously enhance endosomal escape, reduce inflammatory toxicity, and increase reproducibility [129]. Collectively, these refinements are transforming lipid nanoparticles from generic carriers into precision delivery vehicles.

#### 6.1.2 Organ‐Specific Delivery

One of the most significant frontiers in mRNA therapeutics is organ‐selective delivery, in which nanoparticle composition is tuned to preferentially accumulate in defined tissues. This concept recognizes that altering lipid ratios, helper components, particle charge, and physicochemical characteristics can substantially modify in vivo biodistribution [115].

Liver targeting remains the most clinically advanced example due to natural nanoparticle uptake pathways. However, emerging systems are being developed for delivery to the lungs, spleen, bone marrow, heart, skeletal muscle, and central nervous system. Pulmonary targeting may enable treatment of cystic fibrosis, inflammatory lung disease, or respiratory infections. Cardiac targeting could support post‐infarction repair or vascular regeneration. Immune organ targeting may improve vaccine performance and immunotherapy [130].

Central nervous system delivery remains particularly challenging because of the blood–brain barrier. Nevertheless, novel nanoparticles, receptor‐mediated transport systems, intrathecal administration routes, and biomimetic carriers are being explored to enable neurological applications. If successful, organ‐specific delivery would greatly expand the therapeutic scope of mRNA medicine [104].

Beyond lipid nanoparticles, alternative systems such as polymeric nanoparticles, hybrid nanocarriers, extracellular vesicles, and biodegradable micelles are also under active investigation. These platforms may offer unique advantages in stability, repeat dosing, or tissue penetration [124].

### 6.2 mRNA Engineering Improvements

The biological performance of an mRNA therapeutic is determined not only by where it is delivered, but also by how the RNA molecule itself is designed. Modern mRNA engineering integrates principles of molecular biology, structural biophysics, immunology, and computational optimization to maximize protein expression while minimizing unwanted host responses [131].

#### 6.2.1 Codon Optimization

Codon optimization is among the most widely used strategies for enhancing translation efficiency. Because multiple codons can encode the same amino acid, sequence design can be modified to favor codons more efficiently recognized by host tRNA pools, thereby accelerating ribosomal elongation and increasing protein output [132].

However, modern codon optimization extends beyond simple codon frequency replacement. It also considers GC content, RNA secondary structure, ribosome pausing dynamics, motif avoidance, and sequence context near translation initiation sites. Excessive optimization may impair proper protein folding or alter co‐translational processing, meaning balanced design is essential [133].

Optimization of untranslated regions (UTRs) is equally important. Carefully selected 5′ and 3′ UTR sequences can enhance ribosome recruitment, stabilize transcripts, prolong cytoplasmic half‐life, and improve translational consistency. Poly(A) tail length and cap structure engineering further contribute to expression efficiency [134]. Together, these sequence‐level refinements substantially improve potency without increasing dose.

#### 6.2.2 Reduced Immunogenicity

One of the historic limitations of synthetic RNA therapeutics was excessive activation of innate immune pathways. Exogenous RNA can be recognized by pattern recognition receptors such as Toll‐like receptors, RIG‐I‐like receptors, and other cytosolic sensors, triggering inflammatory cytokine production and translational suppression [31].

A major breakthrough in the field was the introduction of modified nucleosides, including pseudouridine and N1‐methyl‐pseudouridine, which reduce innate immune recognition while enhancing translational performance. These modifications were instrumental in enabling clinically successful first‐generation mRNA vaccines and remain foundational to many therapeutic programs [135].

Additional strategies to reduce immunogenicity include removal of double‐stranded RNA contaminants formed during in vitro transcription, purification of full‐length transcripts, optimization of sequence motifs that activate innate sensors, and use of biodegradable carrier materials with improved tolerability. The goal is not necessarily complete immune silence, since some indications benefit from immunostimulation, but rather precise control of immune activation according to therapeutic context [136].

Emerging innovations also include circular RNA constructs, self‐amplifying RNA systems, programmable untranslated regions, and machine learning‐guided sequence design. These approaches may further improve stability, translation duration, and therapeutic index.

### 6.3 Combination Therapies

As the field matures, mRNA therapeutics are increasingly being integrated into combination treatment strategies rather than developed solely as stand‐alone interventions. This reflects a broader recognition that many complex diseases are biologically multifactorial and may respond best to synergistic modalities targeting complementary pathways.

#### 6.3.1 mRNA Plus Small Molecules

Combining mRNA therapeutics with conventional small‐molecule drugs offers several strategic advantages. Small molecules can rapidly modulate signaling pathways, suppress inflammation, alter metabolism, or sensitize tissues to protein‐based interventions, while mRNA provides programmable production of therapeutic proteins or antigens.

In oncology, small molecules that modulate the tumor microenvironment may enhance responsiveness to mRNA cancer vaccines. In metabolic disease, mRNA‐encoded enzymes may be paired with substrate‐lowering drugs to improve biochemical correction. In inflammatory disorders, transient mRNA expression of anti‐inflammatory proteins may complement targeted kinase inhibitors or receptor antagonists [137, 138].

This combination model allows investigators to exploit the rapid pharmacodynamics of small molecules together with the biological specificity of RNA‐directed protein synthesis.

#### 6.3.2 mRNA Plus Immunotherapy

One of the most clinically advanced combination strategies involves mRNA therapeutics with immunotherapy. In cancer treatment, mRNA vaccines can expand tumor‐specific T‐cell repertoires, while checkpoint inhibitors release inhibitory brakes imposed by PD‐1/PD‐L1 or CTLA‐4 pathways. This mechanistic complementarity may generate deeper and more durable antitumor responses than either modality alone [139].

mRNA can also be used to encode cytokines, co‐stimulatory ligands, bispecific antibodies, or immune‐modulating proteins delivered locally or systemically. Such approaches may reprogram immunologically “cold” tumors into inflamed, treatment‐responsive microenvironments [140].

Beyond oncology, mRNA‐immunotherapy combinations may be relevant in chronic viral infection, autoimmune disease, transplantation tolerance, and vaccine enhancement. The programmable nature of mRNA makes it especially attractive for rapidly iterating combination regimens based on evolving biomarker data.

## 7 Challenges and Bottlenecks

Despite the remarkable progress of messenger RNA therapeutics and their rapid transition from experimental platforms to clinically validated interventions, several scientific, manufacturing, economic, and global health barriers continue to constrain their full potential. The success of first‐generation mRNA vaccines demonstrated the feasibility of large‐scale RNA medicine; however, broader therapeutic expansion into chronic diseases, precision oncology, regenerative medicine, and rare disorders requires solutions to persistent bottlenecks that extend beyond proof‐of‐concept efficacy. These challenges include uncertainties regarding long‐term safety, risks of unintended immune activation, high development and production costs, and unequal global access to manufacturing capacity and finished products [7, 141].

Importantly, many of these limitations are not unique to mRNA technologies alone but are amplified by the relative novelty, technical complexity, and specialized infrastructure associated with RNA‐based therapeutics. As the field matures, addressing these barriers will be as critical as molecular innovation itself. The long‐term success of mRNA medicine will depend not only on scientific efficacy, but also on affordability, safety confidence, equitable deployment, and sustainable integration into global healthcare systems [142].

### 7.1 Long‐Term Safety Concerns

Although mRNA therapeutics are generally considered favorable from a safety perspective because they are non‐integrating and transiently expressed, long‐term safety evaluation remains an important priority, particularly as the field expands into chronic or repeat‐dose indications. Most early clinical experience has been derived from prophylactic vaccination, where treatment duration is limited and exposure is episodic. In contrast, applications such as protein replacement therapy, autoimmune modulation, oncology maintenance treatment, or regenerative medicine may require repeated administration over months or years [62, 143].

Under these conditions, cumulative biological effects must be carefully considered. Recurrent exposure to delivery systems, especially lipid nanoparticle formulations, may influence tissue tolerance, biodistribution patterns, hepatic handling, complement activation, or immunogenicity over time. Persistent inflammatory microresponses, although clinically silent in the short term, may become more relevant during chronic administration schedules. Furthermore, repeated induction of potent biological proteins, cytokines, or growth factors requires careful dose control to avoid prolonged pharmacodynamic consequences [69].

Another key issue involves patient heterogeneity. Safety outcomes may differ according to age, comorbidities, genetic background, pregnancy status, immune competence, or underlying inflammatory disease. Populations not extensively represented in early clinical trials may require dedicated post‐marketing surveillance and real‐world evidence programs [144].

Long‐term pharmacovigilance systems, registry‐based monitoring, and mechanistic biomarker studies will therefore remain essential. Establishing robust safety confidence is particularly important for therapeutic indications where patients may receive repeated or lifelong treatment rather than one‐time preventive intervention.

### 7.2 Off‐Target Immune Activation

The ability of RNA molecules to interact with the innate immune system is both an advantage and a challenge. In vaccine settings, controlled activation of innate immune pathways can function as a built‐in adjuvant, enhancing antigen presentation and adaptive immune priming. However, in many non‐vaccine therapeutic applications, unintended immune stimulation may reduce efficacy or increase adverse effects [31].

Exogenous RNA can be recognized by cellular pattern recognition receptors, including endosomal Toll‐like receptors and cytosolic RNA sensors, leading to induction of type I interferons, inflammatory cytokines, and antiviral defense programs. While these pathways are biologically protective, excessive activation can suppress translation of the therapeutic mRNA, shorten expression duration, and contribute to systemic reactogenicity [101].

Off‐target immune activation may also arise from impurities generated during manufacturing, particularly double‐stranded RNA byproducts formed during in vitro transcription. Even low levels of such contaminants can alter innate immune responses and reduce product consistency. Similarly, certain lipid or polymeric carriers may activate complement pathways or inflammatory signaling independent of the mRNA cargo itself [145].

The challenge is therefore not simply to eliminate immunogenicity, but to calibrate it according to therapeutic need. Vaccines may benefit from partial immune stimulation, whereas protein replacement or regenerative applications often require immune quiescence. Achieving this balance depends on continued progress in nucleoside modification, purification technologies, sequence engineering, and biocompatible delivery systems [38].

### 7.3 Cost and Accessibility

Despite the speed and flexibility of mRNA manufacturing relative to some traditional biologics, the current economic profile of many RNA therapeutics remains a substantial barrier to widespread adoption. High costs arise from multiple sources, including specialized raw materials, proprietary lipid components, nucleoside‐modified substrates, sterile manufacturing environments, cold‐chain logistics, advanced quality testing, and intellectual property constraints [146].

For personalized applications such as individualized cancer vaccines, costs may increase further because each patient‐specific product requires tumor sequencing, bioinformatic antigen selection, rapid custom synthesis, release testing, and time‐sensitive clinical administration. These complex workflows challenge conventional reimbursement systems and may limit access to patients in high‐resource settings only [92].

Rare disease therapies face related economic difficulties. Although mRNA platforms may lower some development barriers compared with bespoke biologics or viral gene therapies, the small patient populations involved can still result in high per‐patient pricing. Without innovative financing mechanisms, many patients may remain excluded from treatment [72].

Cost also influences health system adoption. Even clinically effective products may face delayed uptake if procurement budgets, insurance coverage, or public reimbursement pathways are inadequate. As mRNA medicine expands, economic sustainability will become increasingly important alongside scientific performance [57].

Strategies to reduce cost include manufacturing scale efficiencies, standardized platform processes, modular production facilities, thermostable formulations that lower logistics expenses, and competitive market entry by multiple developers. Over time, these measures may significantly improve affordability.

### 7.4 Global Distribution Inequities

The unequal global distribution of advanced therapeutics remains one of the most significant ethical and public health challenges in modern medicine, and mRNA technologies are no exception. The rapid deployment of mRNA vaccines highlighted striking disparities in manufacturing ownership, procurement power, supply chain control, and access between high‐income and low‐income regions [147].

A limited number of countries currently possess the infrastructure necessary for large‐scale GMP RNA production, nanoparticle formulation, fill‐finish operations, and ultra‐cold logistics. As a result, many low‐ and middle‐income countries remain dependent on imports, donations, or delayed procurement cycles. This dependence can create vulnerability during periods of global demand surge or geopolitical disruption [148].

Distribution inequities are not solely logistical; they are also technological. Limited local capacity for formulation science, quality control analytics, regulatory review, and workforce training can delay regional adoption even when products are available [149]. Intellectual property barriers, technology transfer limitations, and concentration of manufacturing expertise further widen the gap.

If mRNA therapeutics become important treatments for cancer, rare diseases, cardiovascular disorders, or chronic inflammatory conditions, unequal access could extend beyond pandemics into long‐term structural healthcare disparities. Addressing this risk requires deliberate global strategy rather than reactive crisis response [148].

Priority solutions include regional manufacturing hubs, voluntary licensing frameworks, local workforce development, harmonized regulatory support, thermostable product design, and investment in resilient supply chains across Africa, Latin America, Asia, and other underserved regions. Equitable access should be considered a core component of innovation rather than an afterthought [150].

### 7.5 Integrated Perspective on Future Bottlenecks

The challenges facing mRNA therapeutics are interconnected rather than isolated. Safety uncertainty can increase regulatory burden; regulatory complexity can raise costs; high costs can worsen access inequities; and limited access can reduce real‐world evidence generation in diverse populations. Similarly, inadequate delivery systems may require higher doses, increasing both expense and reactogenicity.

Consequently, overcoming bottlenecks will require systems‐level solutions that integrate molecular engineering, manufacturing innovation, health economics, regulatory science, and global policy. Scientific progress alone will not guarantee successful therapeutic adoption [6, 151].

## 8 Future Perspectives

The remarkable progress of messenger RNA therapeutics over the past decade has established the platform as one of the most dynamic innovations in contemporary medicine. What began as a promising but technically constrained nucleic acid strategy has rapidly evolved into a clinically validated modality with expanding applications across vaccination, oncology, rare disease treatment, and protein replacement therapy. Yet the current generation of mRNA products likely represents only the earliest phase of a much broader therapeutic transformation. Future advances are expected to move beyond generalized platform deployment toward highly individualized, data‐driven, and disease‐specific applications integrated within precision healthcare systems [92, 107].

Several converging forces are likely to shape this next era. Improvements in delivery science, synthetic RNA engineering, systems biology, computational modeling, and advanced manufacturing are creating opportunities for more precise and durable therapeutic interventions. At the same time, increasing understanding of disease heterogeneity is driving demand for treatments tailored to individual genetic, molecular, and immunological profiles. In this context, mRNA is uniquely positioned because its encoded sequence can be rapidly redesigned without fundamentally changing the production framework [103].

The future of mRNA medicine is therefore expected to be defined not simply by new products, but by a new therapeutic paradigm in which programmable biological instructions can be customized, updated, and deployed with unprecedented speed.

### 8.1 Personalized and Precision mRNA Medicine

One of the most transformative future directions for mRNA therapeutics is the emergence of personalized and precision medicine. Conventional pharmaceuticals are typically developed for broad patient populations, often with variable efficacy due to genetic diversity, disease heterogeneity, and differential drug metabolism. In contrast, mRNA therapeutics offer a modular platform in which the encoded sequence can be individualized while preserving a common manufacturing backbone [2].

This capability is already most visible in oncology, where personalized mRNA cancer vaccines can be designed using patient‐specific tumor sequencing data. Somatic mutations unique to an individual tumor generate neoantigens that can be computationally identified and incorporated into customized mRNA constructs to stimulate targeted immune responses. Such strategies may significantly improve specificity while reducing damage to healthy tissues [94].

Beyond cancer, precision mRNA medicine may become relevant for inherited disorders caused by unique pathogenic variants, enzyme deficiencies requiring individualized protein design, or rare diseases affecting small patient populations traditionally neglected by conventional drug development. Because mRNA products can be rapidly redesigned, they may also enable adaptive treatment approaches in which therapy evolves in response to disease progression or biomarker changes.

Integration with genomic medicine, companion diagnostics, and longitudinal patient monitoring is likely to accelerate this transition [2]. In the future, therapeutic selection may involve matching patients not only to a drug class, but to a sequence‐optimized RNA medicine tailored to their molecular profile.

### 8.2 Artificial Intelligence and AI‐Driven Antigen Design

Artificial intelligence (AI) is poised to become a major accelerator of mRNA therapeutic development. Designing effective mRNA medicines requires optimization across multiple parameters, including antigen selection, codon usage, RNA secondary structure, immunogenic motifs, untranslated regions, delivery compatibility, and manufacturability [152]. These multidimensional problems are highly suitable for machine learning and predictive modeling.

In vaccine development, AI‐driven antigen design can rapidly identify immunogenic epitopes, conserved pathogen targets, and mutation‐resistant antigen combinations. For cancer immunotherapy, machine learning models can improve neoantigen prediction by integrating genomic mutations [153], peptide processing, HLA binding affinity, and T‐cell recognition probability. This may substantially enhance the efficacy of personalized cancer vaccines.

AI is also expected to improve the mRNA molecule itself. Predictive algorithms can optimize codon selection, minimize undesirable secondary structures, reduce innate immune activation motifs, and enhance translation efficiency. In delivery science, computational approaches may accelerate discovery of novel lipid chemistries and nanoparticle formulations with superior organ specificity and tolerability [152].

More broadly, AI may shorten development timelines by integrating preclinical, manufacturing, and clinical datasets to predict responders, refine dosing strategies, and identify safety signals earlier. The combination of programmable RNA therapeutics with AI‐guided design could create a rapid‐response medical ecosystem capable of iterating treatments in near real time.

### 8.3 Expansion Into Chronic Disease Management

To date, much of the public visibility of mRNA technology has centered on vaccines and acute interventions. However, one of the most important long‐term opportunities lies in the management of chronic diseases, which account for a major proportion of global morbidity and healthcare expenditure [154].

For chronic metabolic disorders, mRNA therapeutics may enable repeated replacement of deficient enzymes, transport proteins, or regulatory hormones. In cardiovascular disease, periodic administration of mRNA encoding reparative or angiogenic factors may support tissue recovery and vascular function. In inflammatory and autoimmune conditions, mRNA constructs designed to induce immune tolerance or deliver anti‐inflammatory mediators could offer targeted alternatives to lifelong immunosuppression [155].

Chronic pulmonary diseases such as cystic fibrosis or inherited respiratory disorders may benefit from inhaled mRNA therapies delivering functional proteins directly to affected tissues. Similarly, recurrent dosing strategies for neurodegenerative disease may provide neuroprotective factors or disease‐modifying proteins if delivery barriers can be overcome.

Expansion into chronic disease management will require advances beyond those needed for vaccines. Key priorities include improved repeat‐dose tolerability, longer expression duration, reduced immunogenicity, patient‐friendly administration routes, and economically sustainable treatment models. Success in these areas would greatly expand the medical relevance of mRNA therapeutics from episodic care to continuous disease management [87].

### 8.4 Potential Role in Regenerative Medicine

Regenerative medicine represents another highly promising frontier for mRNA therapeutics. Unlike permanent gene transfer strategies, mRNA enables transient expression of regenerative proteins, making it particularly attractive when temporary biological signaling is sufficient to stimulate repair processes [62].

In cardiovascular medicine, mRNA encoding angiogenic factors, survival proteins, or tissue repair mediators may enhance recovery following myocardial infarction or ischemic injury. In musculoskeletal medicine, mRNA delivery of growth factors could support bone healing, cartilage regeneration, tendon repair, or skeletal muscle recovery after trauma [81].

mRNA is also being explored in stem cell engineering and ex vivo cell programming. Temporary delivery of transcription factors via mRNA can reprogram cell fate without genomic integration, reducing safety concerns associated with viral vectors [156]. This strategy may be valuable in generating induced pluripotent stem cells, engineered immune cells, or lineage‐specific regenerative cell products.

Wound healing, dermatologic repair, liver regeneration, and organ fibrosis reversal are additional areas where transient, localized protein expression may offer therapeutic benefit. Because regeneration often requires tightly timed signaling rather than permanent genetic alteration, mRNA therapeutics are conceptually well matched to this field [157].

### 8.5 Toward an Integrated mRNA Therapeutic Ecosystem

The future trajectory of mRNA medicine is likely to involve convergence rather than isolated innovation. Personalized sequencing data, AI‐guided design, automated manufacturing, smart delivery systems, and real‐world clinical analytics may become integrated into a unified therapeutic ecosystem. In such a model, diagnosis, molecular profiling, RNA sequence generation, production, and treatment deployment could occur on accelerated timelines unimaginable in conventional pharmaceutical development [158].

Hospitals or regional production centers may eventually manufacture customized mRNA therapeutics locally under standardized quality frameworks. Digital health tools could guide dosing intervals based on biomarker response [159]. Combination therapies may pair mRNA medicines with biologics, cell therapies, or genome editing according to patient need.

This systems‐level integration would redefine the relationship between diagnosis and treatment, allowing medicine to become more responsive, adaptive, and personalized.

## 9 Conclusion

Messenger RNA therapeutics have rapidly progressed from an experimental nucleic acid concept to one of the most transformative biomedical platforms of the modern era. Advances in RNA chemistry, sequence engineering, delivery technologies, and scalable manufacturing have collectively redefined the therapeutic potential of transient gene expression. What was once limited by instability, inefficient intracellular delivery, and excessive innate immune activation has now matured into a clinically validated modality capable of addressing diverse medical needs. The successful deployment of mRNA vaccines during the COVID‐19 pandemic provided decisive real‐world confirmation that synthetic RNA medicines can be designed rapidly, manufactured at global scale, and deployed safely with substantial public health impact.

Beyond infectious disease prevention, the field has entered a second and more expansive phase characterized by diversification into therapeutic medicine. mRNA platforms are now being actively developed for cancer immunotherapy, personalized neoantigen vaccines, immune tolerance induction in autoimmune disease, protein replacement for inherited disorders, cardiovascular regeneration, neurological applications, and rare genetic conditions. This breadth of applicability reflects a defining strength of mRNA technology: the encoded sequence can be modified to generate fundamentally different therapeutic products while preserving a common manufacturing and delivery framework. Few therapeutic modalities offer comparable flexibility, speed of redesign, and translational scalability.

Several key advances have driven this progress. Lipid nanoparticle systems and emerging targeted carriers have substantially improved intracellular delivery and biodistribution. Modified nucleosides, codon optimization, refined untranslated regions, and advanced purification methods have enhanced translational efficiency while reducing unwanted immunogenicity. Simultaneously, increasing integration of computational biology, biomarker science, and artificial intelligence is enabling more rational antigen selection, improved sequence design, and accelerated development pipelines. These innovations are progressively converting mRNA therapeutics from first‐generation products into precision‐engineered medicines.

Despite this momentum, important challenges remain before widespread clinical integration can be fully realized. Long‐term safety monitoring, repeat‐dose tolerability, tissue‐specific delivery, cold‐chain dependence, manufacturing cost, and equitable global access continue to represent major translational priorities. Regulatory frameworks must also evolve to accommodate personalized products, rapid sequence updates, decentralized manufacturing, and increasingly complex combination therapies. The next phase of progress will therefore require not only scientific innovation, but also coordinated advances in health systems, policy, regulatory science, and global manufacturing capacity.

Looking forward, the transformative potential of mRNA therapeutics extends well beyond the current pipeline. The platform is uniquely positioned to support personalized medicine, rapid outbreak response, chronic disease management, regenerative therapies, and adaptive treatment models guided by real‐time molecular data. As delivery systems become more precise and production systems more accessible, mRNA medicines may increasingly move from specialized interventions to routine components of clinical care.

In conclusion, mRNA therapeutics represent more than a new drug class; they signify a fundamental shift toward programmable medicine. Their capacity to deliver transient biological instructions with speed, precision, and versatility places them among the most promising healthcare technologies of the 21st century. Continued interdisciplinary innovation and responsible global implementation will determine how fully this promise is realized, but the trajectory is clear: mRNA is poised to become a foundational pillar of future medicine.

## Author Contributions

**Gedion Mengistu Dejen:** conceptualization, writing – original draft, writing – review and editing, software.

## Funding

The author has nothing to report.

## Conflicts of Interest

The author declares no conflicts of interest.

## Use of Artificial Intelligence Tools

AI‐assisted tools were not used to generate scientific content, interpret the literature, analyze data, or develop the scientific conclusions. The author assumes full responsibility for the accuracy, integrity, originality, and final content of the manuscript.

## Data Availability

The author has nothing to report.

## References

[1] K. Y. Leong , S. K. Tham , and C. L. Poh , “Revolutionizing Immunization: A Comprehensive Review of mRNA Vaccine Technology and Applications,” Virology Journal 22, no. 1 (2025): 71, 10.1186/s12985-025-02645-6. PMC11900334 40075519

[2] M. A. Ige , X. Ren , Y. Yang , et al., “mRNA Therapeutics: Transforming Medicine Through Innovation in Design, Delivery, and Disease Treatment,” Molecular Therapy Nucleic Acids 36, no. 4 (2025): 102721, 10.1016/j.omtn.2025.102721. PMC12590236 41210584

[3] K. Batisani , “The Role of mRNA Vaccines in Infectious Diseases: A New Era of Immunization,” Tropical Diseases, Travel Medicine and Vaccines 11 (2025): 12, 10.1186/s40794-025-00246-3. PMC12079950 40369626

[4] J. Li , Y. Liu , J. Dai , et al., “mRNA Vaccines: Current Applications and Future Directions,” MedComm 6, no. 11 (2025): e70434, 10.1002/mco2.70434. PMC12572956 41179708

[5] D. Haghmorad , M. Eslami , N. Orooji , et al., “mRNA Vaccine Platforms: Linking Infectious Disease Prevention and Cancer Immunotherapy,” Frontiers in Bioengineering and Biotechnology 13 (2025): 1547025, 10.3389/fbioe.2025.1547025. PMC11937095 40144393

[6] A. Wagh , S. Jaiswal , and D. Bornare , “A Review: Extraction of Essential Oil From Lemon Grass as a Preservative for Animal Products,” Journal of Pharmacognosy and Phytochemistry 10 (2021): 26–31, 10.22271/phyto.2021.v10.i3Sa.14187.

[7] S. K. Makkar , “Advances in RNA‐Based Therapeutics: Current Breakthroughs, Clinical Translation, and Future Perspectives,” Frontiers in Genetics 16 (2025): 1675209, 10.3389/fgene.2025.1675209. PMC12592931 41211454

[8] S. Saxena , V. Mandrah , W. Tariq , P. Das , K. Sambhav , and S. H. Devi , “The Future of mRNA Vaccines: Potential Beyond COVID‐19,” Cureus 17, no. 5 (2025): e84529, 10.7759/cureus.84529. PMC12179814 40546533

[9] J. Ma , M. Li , Z. Xie , and D. Li , “mRNA Vaccination Facilitates the Prevention and Control of Infectious Diseases at an Unprecedented Speed,” Decoding Infection and Transmission 3 (2025): 100048, 10.1016/j.dcit.2025.100048.

[10] P. Garg , R. Salgia , and S. S. Singhal , “mRNA‐Based Cancer Vaccines: A New Frontier in Personalized Immunotherapy,” Biochimica et Biophysica Acta, Reviews on Cancer 1881, no. 3 (2026): 189577, 10.1016/j.bbcan.2026.189577. 41861922

[11] R. Gu , D. Si , D. Lei , X. Xie , Y. Li , and H. Wen , “Accelerate the Highly Efficient Development of mRNA Vaccines Through Advanced Computational Methods,” MedComm (2020) 7, no. 2 (2026): e70612, 10.1002/mco2.70612. PMC12883036 41664706

[12] V. H. Cowling , “Regulation of mRNA cap Methylation,” Biochemical Journal 425, no. pt. 2 (2010): 295–302, 10.1042/BJ20091352. PMC2825737 20025612

[13] A. Ely , P. Singh , T. S. Smith , and P. Arbuthnot , “In Vitro Transcribed mRNA for Expression of Designer Nucleases: Advantages as a Novel Therapeutic for the Management of Chronic HBV Infection,” Advanced Drug Delivery Reviews 168 (2021): 134–146, 10.1016/j.addr.2020.05.010. 32485207

[14] D. N. Antropov , O. V. Markov , A. S. Dome , et al., “A New Combination of 5′‐ and 3′‐Untranslated Regions Increases the Expression of mRNA In Vitro and In Vivo,” Vavilovskii Zh Genet Selektsii 29, no. 6 (2025): 737–743, 10.18699/vjgb-25-81. PMC12559688 41164277

[15] L. A. Passmore and J. Coller , “Roles of mRNA poly(A) Tails in Regulation of Eukaryotic Gene Expression,” Nature Reviews Molecular Cell Biology 23, no. 2 (2022): 93–106, 10.1038/s41580-021-00417-y. PMC7614307 34594027

[16] M. Bérouti , M. Wagner , W. Greulich , et al., “Pseudouridine RNA Avoids Immune Detection Through Impaired Endolysosomal Processing and TLR Engagement,” Cell 188, no. 18 (2025): 4880–4895.e15, 10.1016/j.cell.2025.05.032. 40580950

[17] Y. Mei and X. Wang , “RNA Modification in mRNA Cancer Vaccines,” Clinical and Experimental Medicine 23 (2023): 1–15, 10.1007/s10238-023-01020-5. PMC9928499 36788153

[18] H. Zhou , D. Wei , Z. Chen , et al., “The mRNA‐Based Innovative Strategy: Progress and Challenges,” Nano‐Micro Letters 18 (2026): 118, 10.1007/s40820-025-01906-x. PMC12804527 41535643

[19] E. M. Chelan , A. Parhizkar , M. Nemati , et al., “Targeted Cytosolic Delivery of mRNA Immunotherapeutics: From Vaccine Delivery to Protein Replacement,” Biomedicine and Pharmacotherapy 198 (2026): 119181, 10.1016/j.biopha.2026.119181. 41856071

[20] Nitika , J. Wei , and A. M. Hui , “The Delivery of mRNA Vaccines for Therapeutics,” Life (Basel) 12, no. 8 (2022): 1254, 10.3390/life12081254. PMC9410089 36013433

[21] T. Ikrar , W. Muchsin , and A. Sophian , “mRNA Vaccine Platforms and Lipid Nanoparticle Delivery Systems: Molecular Advances, Clinical Breakthroughs, and Regulatory Perspectives (2020–2025)” [Internet]. Research Square, 2026, accessed April 21, 10.21203/rs.3.rs-9109248/v1.

[22] K. Swetha , N. G. Kotla , L. Tunki , et al., “Recent Advances in the Lipid Nanoparticle‐Mediated Delivery of mRNA Vaccines,” Vaccines (Basel) 11, no. 3 (March 2023): 658, 10.3390/vaccines11030658. PMC10059764 36992242

[23] A. E. Laturski , M. T. Dulay , J. L. Perry , and J. M. DeSimone , “Transfection via RNA‐Based Nanoparticles: Comparing Encapsulation vs Adsorption Approaches of RNA Incorporation,” Bioconjugate Chemistry 36, no. 3 (March 2025): 367–376, 10.1021/acs.bioconjchem.5c00028. 39999074

[24] M. Dalabehera , A. Ghosh , S. Mohanty , et al., “mRNA Delivery Systems 2.0: Engineering Extrahepatic Delivery for Non‐Vaccine Therapeutics,” Materials Today Bio 35 (December 2025): 102584, 10.1016/j.mtbio.2025.102584. PMC12719227 41438702

[25] C. Forenzo , N. Arnold , and J. Larsen , “Emerging Polymeric Nanocarriers for mRNA and Protein Therapeutics: Design, Challenges, and Clinical Outlook,” Nanomedicine (Lond) 20, no. 18 (September 2025): 2357–2374, 10.1080/17435889.2025.2542110. PMC12413044 40762456

[26] H. O. Alsaab , F. D. Alharbi , A. S. Alhibs , et al., “PLGA‐Based Nanomedicine: History of Advancement and Development in Clinical Applications of Multiple Diseases,” Pharmaceutics 14, no. 12 (December 2022): 2728, 10.3390/pharmaceutics14122728. PMC9786338 36559223

[27] M. Taghdiri and C. Mussolino , “Viral and Non‐Viral Systems to Deliver Gene Therapeutics to Clinical Targets,” International Journal of Molecular Sciences 25, no. 13 (July 2024): 7333, 10.3390/ijms25137333. PMC11242246 39000440

[28] G. Geng , Y. Xu , Z. Hu , et al., “Viral and Non‐Viral Vectors in Gene Therapy: Current State and Clinical Perspectives,” EBioMedicine 118 (July 2025): 105834, 10.1016/j.ebiom.2025.105834. PMC12271757 40602323

[29] Y. Liu , Y. Huang , G. He , C. Guo , J. Dong , and L. Wu , “Development of mRNA Lipid Nanoparticles: Targeting and Therapeutic Aspects,” International Journal of Molecular Sciences 25, no. 18 (September 2024): 10166, 10.3390/ijms251810166. PMC11432440 39337651

[30] W. Cao and T. Xia , “Optimized Strategies for Precise Messenger RNA Delivery Using Site‐Specific Lipid Nanoparticle‐Based Drug Delivery Systems,” Nanomedicine (Lond) 20, no. 1 (2024): 1–4, 10.1080/17435889.2024.2406218. PMC11703114 39345133

[31] R. Verbeke , M. J. Hogan , K. Loré , and N. Pardi , “Innate Immune Mechanisms of mRNA Vaccines,” Immunity 55, no. 11 (November 2022): 1993–2005, 10.1016/j.immuni.2022.10.014. PMC9641982 36351374

[32] E. Bettini and M. Locci , “SARS‐CoV‐2 mRNA Vaccines: Immunological Mechanism and Beyond,” Vaccines (Basel) 9, no. 2 (February 2021): 147, 10.3390/vaccines9020147. PMC7918810 33673048

[33] S. Son and K. Lee , “Development of mRNA Vaccines and Their Delivery System,” Molecules and Cells 46, no. 1 (January 2023): 41–47, 10.14348/molcells.2023.2165. PMC9880606 36697236

[34] X. Hou , J. Shi , and Y. Xiao , “mRNA Medicine: Recent Progresses in Chemical Modification, Design, and Engineering,” Nano Research 17, no. 10 (October 2024): 9015–9030, 10.1007/s12274-024-6978-6. PMC12311907 40756674

[35] I. Mantel , B. A. Sadiq , and J. M. Blander , “Spotlight on TAP and Its Vital Role in Antigen Presentation and Cross‐Presentation,” Molecular Immunology 142 (February 2022): 105–119, 10.1016/j.molimm.2021.12.013. PMC9241385 34973498

[36] P. A. Roche and K. Furuta , “The Ins and Outs of MHC Class II‐Mediated Antigen Processing and Presentation,” Nature Reviews. Immunology 15, no. 4 (April 2015): 203–216, 10.1038/nri3818. PMC6314495 25720354

[37] Y. Quan , H. Yang , W. Li , and L. Li , “mRNA Vaccines: Immunogenicity and Quality Characteristics,” Journal of Nanobiotechnology 24 (November 2025): 6, 10.1186/s12951-025-03800-5. PMC12765321 41310620

[38] A. K. Minnaert , H. Vanluchene , R. Verbeke , et al., “Strategies for Controlling the Innate Immune Activity of Conventional and Self‐Amplifying mRNA Therapeutics: Getting the Message Across,” Advanced Drug Delivery Reviews 176 (September 2021): 113900, 10.1016/j.addr.2021.113900. PMC8325057 34324884

[39] W. Zhou , L. Jiang , S. Liao , et al., “Vaccines' New Era‐RNA Vaccine,” Viruses 15, no. 8 (August 2023): 1760, 10.3390/v15081760. PMC10458896 37632102

[40] H. J. Hwang and Y. K. Kim , “Molecular Mechanisms of Circular RNA Translation,” Experimental & Molecular Medicine 56, no. 6 (June 2024): 1272–1280, 10.1038/s12276-024-01220-3. PMC11263353 38871818

[41] A. Medjmedj , H. Genon , D. Hezili , et al., “Evaluation of Synthetic mRNA With Selected UTR Sequences and Alternative Poly(A) Tail, In Vitro and In Vivo,” Molecular Therapy. Nucleic Acids 36, no. 3 (July 2025): 102648, 10.1016/j.omtn.2025.102648. PMC12355064 40822034

[42] R. A. Wesselhoeft , P. S. Kowalski , and D. G. Anderson , “Engineering Circular RNA for Potent and Stable Translation in Eukaryotic Cells,” Nature Communications 9, no. 1 (July 2018): 2629, 10.1038/s41467-018-05096-6. PMC6035260 29980667

[43] J. D. G. Comes , G. P. Pijlman , and T. A. H. Hick , “Rise of the RNA Machines‐Self‐Amplification in mRNA Vaccine Design,” Trends in Biotechnology 41, no. 11 (June 2023): 1417–1429, 10.1016/j.tibtech.2023.05.007. PMC10266560 37328401

[44] A. A. Deviatkin , R. A. Simonov , K. A. Trutneva , et al., “Cap‐Independent Circular mRNA Translation Efficiency,” Vaccines (Basel) 11, no. 2 (January 2023): 238, 10.3390/vaccines11020238. PMC9967249 36851116

[45] Y. Yang and Z. Wang , “IRES‐Mediated Cap‐Independent Translation, a Path Leading to Hidden Proteome,” Journal of Molecular Cell Biology 11, no. 10 (September 2019): 911–919, 10.1093/jmcb/mjz091. PMC6884710 31504667

[46] T. Nagashima , N. Inoue , N. Yumoto , et al., “Feedforward Regulation of mRNA Stability by Prolonged Extracellular Signal‐Regulated Kinase Activity,” FEBS Journal 282, no. 4 (February 2015): 613–629, 10.1111/febs.13172. PMC4334673 25491268

[47] M. M. Janjic , R. M. Prévide , P. A. Fletcher , et al., “Divergent Expression Patterns of Pituitary Gonadotropin Subunit and GnRH Receptor Genes to Continuous GnRH In Vitro and In Vivo,” Scientific Reports 9, no. 1 (December 2019): 20098, 10.1038/s41598-019-56480-1. PMC6934515 31882740

[48] B. Eiz‐Vesper and H. M. Schmetzer , “Antigen‐Presenting Cells: Potential of Proven Und New Players in Immune Therapies,” Transfusion Medicine and Hemotherapy: Offizielles Organ Der Deutschen Gesellschaft Fur Transfusionsmedizin Und Immunhamatologie 47, no. 6 (December 2020): 429–431, 10.1159/000512729. PMC7768096 33442337

[49] S. Oloruntimehin , F. Akinyi , M. Paul , and O. Ariyo , “mRNA Vaccine Technology Beyond COVID‐19,” Vaccines (Basel) 13, no. 6 (May 2025): 601, 10.3390/vaccines13060601. PMC12197385 40573932

[50] J. A. Wojcechowskyj , R. M. Jong , I. Mäger , et al., “Controlling Reactogenicity While Preserving Immunogenicity From a Self‐Amplifying RNA Vaccine by Modulating Nucleocytoplasmic Transport,” NPJ Vaccines 10, no. 1 (April 2025): 85, 10.1038/s41541-025-01135-8. PMC12041602 40301369

[51] A. K. Blakney , S. Ip , and A. J. Geall , “An Update on Self‐Amplifying mRNA Vaccine Development,” Vaccines (Basel) 9, no. 2 (January 2021): 97, 10.3390/vaccines9020097. PMC7911542 33525396

[52] U. Okechukwu Paul‐Chima , O. Michael Ben , C. O. Fabian , U. Jovita Nnenna , and N. U. Chinyere , “Self‐Amplifying RNA (saRNA) and Circular RNA (circRNA) Vaccines: Progress, Evidence Gaps, and Translational Pathways for Durable and Scalable Immunization,” Human Vaccines & Immunotherapeutics 22, no. 1 (December 2026): 2661120, 10.1080/21645515.2026.2661120. PMC13108351 42015917

[53] S. Khorshid Sokhangouy , M. Behzadi , S. Rezaei , et al., “mRNA Vaccines: Design Principles, Mechanisms, and Manufacturing‐Insights From COVID‐19 as a Model for Combating Infectious Diseases,” Biotechnology Journal 20, no. 2 (2025): e202400596, 10.1002/biot.202400596. 39989260

[54] X. Liu , Y. Zhang , S. Zhou , L. Dain , L. Mei , and G. Zhu , “Circular RNA: An Emerging Frontier in RNA Therapeutic Targets, RNA Therapeutics, and mRNA,” Journal of Controlled Release 348 (August 2022): 84–94, 10.1016/j.jconrel.2022.05.043. PMC9644292 35649485

[55] Y. Du , P. K. Zuber , H. Xiao , X. Li , Y. Gordiyenko , and V. Ramakrishnan , “Efficient Circular RNA Synthesis for Potent Rolling Circle Translation,” Nature Biomedical Engineering 9, no. 7 (July 2025): 1062–1074, 10.1038/s41551-024-01306-3. PMC12270912 39672985

[56] K. Chen , Y. Xu , J. Li , et al., “The Potential and Challenges of Circular RNA in the Development of Vaccines and Drugs for Emerging Infectious Diseases,” Molecular Therapy. Nucleic Acids 36, no. 3 (August 2025): 102687, 10.1016/j.omtn.2025.102687. PMC12423345 40951762

[57] S. H. Seo and M. K. Song , “Advancements and Challenges in Next‐Generation mRNA Vaccine Manufacturing Systems,” Clinical and Experimental Vaccine Research 14, no. 4 (October 2025): 299, 10.7774/cevr.2025.14.e40. PMC12599430 41221170

[58] A. J. Barbier , A. Y. Jiang , P. Zhang , R. Wooster , and D. G. Anderson . 2022. “The Clinical Progress of mRNA Vaccines and Immunotherapies,” Nature Biotechnology 40, no. 6: 840–854, 10.1038/s41587-022-01294-2. 35534554

[59] N. Al Fayez , M. S. Nassar , A. A. Alshehri , et al., “Recent Advancement in mRNA Vaccine Development and Applications,” Pharmaceutics 15, no. 7 (July 2023): 1972, 10.3390/pharmaceutics15071972. PMC10384963 37514158

[60] V. R. Litvinova , A. P. Rudometov , L. I. Karpenko , and A. A. Ilyichev , “mRNA Vaccine Platform: mRNA Production and Delivery,” Russian Journal of Bioorganic Chemistry 49, no. 2 (2023): 220–235, 10.1134/S1068162023020152. PMC10197051 37252004

[61] S. Qin , X. Tang , Y. Chen , et al., “mRNA‐Based Therapeutics: Powerful and Versatile Tools to Combat Diseases,” Signal Transduction and Targeted Therapy 7, no. 1 (May 2022): 166, 10.1038/s41392-022-01007-w. PMC9123296 35597779

[62] O. Chabanovska , A. M. Galow , R. David , and H. Lemcke , “mRNA—A Game Changer in Regenerative Medicine, Cell‐Based Therapy and Reprogramming Strategies,” Advanced Drug Delivery Reviews 179 (December 2021): 114002, 10.1016/j.addr.2021.114002. PMC9418126 34653534

[63] X. Zhen , W. Chen , and W. Tao , “mRNA‐Based Technology for Engineered Regenerative Medicine,” Cell Biomater 2, no. 1 (January 2026): 100185, 10.1016/j.celbio.2025.100185.

[64] R. E. Young , S. I. Hofbauer , and R. S. Riley , “Overcoming the Challenge of Long‐Term Storage of mRNA‐Lipid Nanoparticle Vaccines,” Molecular Therapy 30, no. 5 (May 2022): 1792–1793, 10.1016/j.ymthe.2022.04.004. PMC9023316 35452599

[65] N. N. Zhang , X. F. Li , Y. Q. Deng , et al., “A Thermostable mRNA Vaccine Against COVID‐19,” Cell 182, no. 5 (September 2020): 1271–1283.e16, 10.1016/j.cell.2020.07.024. PMC7377714 32795413

[66] M. F. H. Khan , F. Baudin , A. Sudalaiyadum Perumal , and A. A. Kamen , “Freeze‐Drying of mRNA‐LNPs Vaccines: A Review,” Vaccines (Basel) 13, no. 8 (August 2025): 853, 10.3390/vaccines13080853. PMC12389932 40872937

[67] J. Lee , M. C. Woodruff , E. H. Kim , and J. H. Nam , “Knife's Edge: Balancing Immunogenicity and Reactogenicity in mRNA Vaccines,” Experimental & Molecular Medicine 55, no. 7 (July 2023): 1305–1313, 10.1038/s12276-023-00999-x. PMC10394010 37430088

[68] C. Delehedde , L. Even , P. Midoux , C. Pichon , and F. Perche , “Intracellular Routing and Recognition of Lipid‐Based mRNA Nanoparticles,” Pharmaceutics 13, no. 7 (July 2021): 945, 10.3390/pharmaceutics13070945. PMC8308975 34202584

[69] J. Wang , Y. Ding , K. Chong , et al., “Recent Advances in Lipid Nanoparticles and Their Safety Concerns for mRNA Delivery,” Vaccines (Basel) 12, no. 10 (October 2024): 1148, 10.3390/vaccines12101148. PMC11510967 39460315

[70] Y. L. Vishweshwaraiah and N. V. Dokholyan , “mRNA Vaccines for Cancer Immunotherapy,” Frontiers in Immunology 13 (December 2022): 1029069, 10.3389/fimmu.2022.1029069. PMC9794995 36591226

[71] X. Li , P. Jabbarzadeh Kaboli , G. Roozitalab , et al., “Next‐Generation Neoantigen mRNA Vaccines: Immuno‐Engineering Strategies for Precision Cancer Immunotherapy,” Cellular Oncology (Dordrecht) 49, no. 2 (April 2026): 72, 10.1007/s13402-026-01199-1. PMC13083697 41984335

[72] U. Oniūnaitė , O. Yilmaz , R. Ugenskiene , and L. Jankauskaite , “Editorial: Rare Diseases: From Basic Science to Clinical Practice and Public Health,” Frontiers in Pediatrics 13 (April 2025): 1579709, 10.3389/fped.2025.1579709. PMC12040922 40309167

[73] J. F. Murphy , “Personalized Vaccines: Unlocking the Next Era of Medical Innovation in Cancer Immunotherapy,” Cancer Screening and Prevention 4, no. 4 (December 2025): 199–205, 10.14218/CSP.2025.00023.

[74] M. Zouali , “Inverse Vaccination for Autoimmune Diseases: Insights Into the Role of B Lymphocytes,” Cells 14, no. 14 (July 2025): 1085, 10.3390/cells14141085. PMC12293635 40710338

[75] F. Yasmeen , R. H. Pirzada , B. Ahmad , B. Choi , and S. Choi , “Understanding Autoimmunity: Mechanisms, Predisposing Factors, and Cytokine Therapies,” International Journal of Molecular Sciences 25, no. 14 (July 2024): 7666, 10.3390/ijms25147666. PMC11277571 39062908

[76] C. Bezzio , C. A. M. Cavalli , G. Franchellucci , et al., “Psoriasis and Inflammatory Bowel Disease: Concomitant IMID or Paradoxical Therapeutic Effect? A Scoping Review on Anti‐IL‐12/23 and Anti‐IL‐23 Antibodies,” Therapeutic Advances in Gastroenterology 17 (November 2024): 17562848241299564, 10.1177/17562848241299564. PMC11580083 39575159

[77] T. Vavilis , E. Stamoula , A. Ainatzoglou , et al., “mRNA in the Context of Protein Replacement Therapy,” Pharmaceutics 15, no. 1 (January 2023): 166, 10.3390/pharmaceutics15010166. PMC9866414 36678793

[78] A. Karadagi , A. G. Cavedon , H. Zemack , et al., “Systemic Modified Messenger RNA for Replacement Therapy in Alpha 1‐antitrypsin Deficiency,” Scientific Reports 10 (April 2020): 7052, 10.1038/s41598-020-64017-0. PMC7184591 32341402

[79] R. S. Vootukuri , S. Gurung , R. Ghosh , P. B. Mills , J. Baruteau , and H. Zhou , “RNA‐Based Therapies for Inherited Metabolic Disorders,” Journal of Inherited Metabolic Disease 49, no. 2 (March 2026): e70150, 10.1002/jimd.70150. PMC12861719 41621836

[80] J. Chen , Y. Hu , Y. Chen , et al., “LNP‐mRNA Vaccine Prevents Type 1 Diabetes in Non‐Obese Diabetes Mice,” Journal of Controlled Release 375 (November 2024): 513–523, 10.1016/j.jconrel.2024.09.020. 39278354

[81] X. Wang , D. H. Wu , and S. E. Senyo , “mRNA Therapy for Myocardial Infarction: A Review of Targets and Delivery Vehicles,” Frontiers in Bioengineering and Biotechnology 10 (November 2022): 1037051, 10.3389/fbioe.2022.1037051. PMC9732118 36507276

[82] V. Anttila , A. Saraste , J. Knuuti , et al., “Synthetic mRNA Encoding VEGF‐A in Patients Undergoing Coronary Artery Bypass Grafting: Design of a Phase 2a Clinical Trial,” Molecular Therapy. Methods & Clinical Development 18 (July 2020): 464–472, 10.1016/j.omtm.2020.05.030. PMC7369517 32728595

[83] L. He and X. Chen , “Cardiomyocyte Induction and Regeneration for Myocardial Infarction Treatment: Cell Sources and Administration Strategies [Internet],” Advanced Healthcare Materials 9, no. 22 (2020): e2001175, 10.1002/adhm.202001175. 33000909

[84] L. Wang , L. Ma , Z. Gao , Y. Wang , and J. Qiu , “Significance of Gene Therapy in Neurodegenerative Diseases,” Frontiers in Neuroscience 19 (May 2025): 1515255, 10.3389/fnins.2025.1515255. PMC12095248 40406043

[85] P. J. L. Schürmann , S. P. E. van Breda Vriesman , J. A. Castro‐Alpízar , et al., “Therapeutic Application of mRNA for Genetic Diseases,” Wiley Interdisciplinary Reviews: Nanomedicine and Nanobiotechnology 17, no. 3 (2025): e70019, 10.1002/wnan.70019. PMC12104968 40415711

[86] K. J , M. M , T. A , et al., “CFTR mRNA‐Based Gene Therapy for Cystic Fibrosis: A Mutation‐Agnostic Strategy to Restore Ion Transport Function,” Current Gene Therapy (March 2026), 10.2174/0115665232413980251103133741. 41931705

[87] S. Rowe , J. Zuckerman , D. Dorgan , et al., “Inhaled mRNA Therapy for Treatment of Cystic Fibrosis: Interim Results of a Randomized, Double‐Blind, Placebo‐Controlled Phase 1/2 Clinical Study,” Journal of Cystic Fibrosis 22, no. 4 (April 2023): 656–664, 10.1016/j.jcf.2023.04.008. PMC10524666 37121795

[88] S. Badawy , V. Gopinath , A. M. D. Espinosa , et al., “Tissue Regenerative Medicine: Clinical Advances, Challenges, and Opportunities,” APL Bioeng 9, no. 4 (December 2025): 040902, 10.1063/5.0296897. PMC12716889 41424914

[89] S. Spence and V. S. Gallicchio , “Mesenchymal Stem Cell Therapy for Chronic Wound Healing,” Journal of Stem Cell Research [Internet] 3, no. 1 (January 2022): 1–13, https://www.genesispub.org/jscr/mesenchymal-stem-cell-therapy-for-chronic-wound-healing.

[90] Y. Gu , J. Duan , N. Yang , Y. Yang , and X. Zhao , “mRNA Vaccines in the Prevention and Treatment of Diseases,” MedComm (2020) 3, no. 3 (August 2022): e167, 10.1002/mco2.167. PMC9409637 36033422

[91] A. S. Anilkumar , S. M. Thomas , and R. Veerabathiran , “Next‐Generation Cancer Vaccines: Targeting Cryptic and Non‐Canonical Antigens for Precision Immunotherapy,” Exploration of Targeted Anti‐Tumor Therapy 6 (September 2025): 1002338, 10.37349/etat.2025.1002338. PMC12464554 41018485

[92] L. Lu , X. Lu , and W. Luo , “Personalized Cancer Vaccines: Current Advances and Emerging Horizons,” Vaccines (Basel) 13, no. 12 (December 2025): 1231, 10.3390/vaccines13121231. PMC12737412 41441697

[93] M. Magoola and S. K. Niazi , “Current Progress and Future Perspectives of RNA‐Based Cancer Vaccines: A 2025 Update,” Cancers (Basel) 17, no. 11 (June 2025): 1882, 10.3390/cancers17111882. PMC12153701 40507360

[94] M. Parganiha , J. Rathored , and D. Sai Painkra , “mRNA Vaccines in Oncology: Personalized Cancer Immunization and Neoantigen Targeting,” Molecular & Cellular Oncology 13, no. 1 (2026): 2652613, 10.1080/23723556.2026.2652613. PMC13064569 41971684

[95] J. Kabut , G. J. Stępień , T. Furgoł , et al., “mRNA Vaccines in Modern Immunotherapy for Non‐Small Cell Lung Cancer (NSCLC)—A Comprehensive Literature Review With Focus on Current Clinical Trials,” Biomedicines 13, no. 9 (September 2025): 2187, 10.3390/biomedicines13092187. PMC12467296 41007751

[96] Y. Song , J. Li , and Y. Wu , “Evolving Understanding of Autoimmune Mechanisms and New Therapeutic Strategies of Autoimmune Disorders,” Signal Transduction and Targeted Therapy 9, no. 1 (October 2024): 263, 10.1038/s41392-024-01952-8. PMC11452214 39362875

[97] R. Razavi , M. Kegel , J. Muscat‐Rivera , D. Weissman , and J. R. Melamed , “Harnessing mRNA‐Lipid Nanoparticles as Innovative Therapies for Autoimmune Diseases,” Molecular Therapy. Methods & Clinical Development 33, no. 3 (September 2025): 101566, 10.1016/j.omtm.2025.101566. PMC12441705 40969676

[98] W. Cao and T. Xia , “mRNA Lipid Nanoparticles Induce Immune Tolerance to Treat Human Diseases,” Medical Review (Berlin) 3, no. 2 (2023): 180–183, 10.1515/mr-2023-0008. PMC10471105 37724087

[99] M. A. Dobrovolskaia , “Pre‐Clinical Immunotoxicity Studies of Nanotechnology‐Formulated Drugs: Challenges, Considerations and Strategy,” Journal of Controlled Release 220, no. pt. B (September 2015): 571, 10.1016/j.jconrel.2015.08.056. PMC4688153 26348388

[100] K. Karikó , H. Muramatsu , F. A. Welsh , et al., “Incorporation of Pseudouridine Into mRNA Yields Superior Nonimmunogenic Vector With Increased Translational Capacity and Biological Stability,” Molecular Therapy 16, no. 11 (September 2008): 1833, 10.1038/mt.2008.200. PMC2775451 18797453

[101] M. Tatematsu , K. Funami , T. Seya , and M. Matsumoto , “Extracellular RNA Sensing by Pattern Recognition Receptors,” Journal of Innate Immunity 10, no. 5–6 (2018): 398–406, 10.1159/000494034. PMC6784046 30404092

[102] Y. Zou , J. M. J. Wong , J. Lin , and Y. Dai , “The Application of Modified mRNA in the Treatment and Prospective Vaccine Development for Cardiovascular Diseases,” Cardiology Plus 10, no. 2 (June 2025): 145, 10.1097/CP9.0000000000000119.

[103] L. M. Gan , M. Lagerström‐Fermér , L. G. Carlsson , et al., “Intradermal Delivery of Modified mRNA Encoding VEGF‐A in Patients With Type 2 Diabetes,” Nature Communications 10 (February 2019): 871, 10.1038/s41467-019-08852-4. PMC6382754 30787295

[104] J. Liu , T. Wang , J. Dong , and Y. Lu , “The Blood‐Brain Barriers: Novel Nanocarriers for Central Nervous System Diseases,” Journal of Nanobiotechnology 23 (February 2025): 146, 10.1186/s12951-025-03247-8. PMC11866817 40011926

[105] M. L. Cacicedo , M. J. Limeres , and S. Gehring , “mRNA‐Based Approaches to Treating Liver Diseases,” Cells 11, no. 20 (October 2022): 3328, 10.3390/cells11203328. PMC9601253 36291194

[106] V. R. Iyer , P. Praveen , B. D. Kaduskar , S. C. Moharir , and R. K. Mishra , “mRNA Biotherapeutics Landscape for Rare Genetic Disorders,” Journal of Biosciences 49, no. 1 (February 2024): 33, 10.1007/s12038-023-00415-6. 38383978

[107] E. Sadaqa , A. A. Fajriawan , and S. E. Muttaqien , “The Evolving Landscape of mRNA Therapeutics: From Molecular Design and Advanced Delivery Systems to Clinical Translation and Future Vistas,” Biointerface Research in Applied Chemistry 16, no. 1 (February 2026): 28, 10.33263/BRIAC161.028.

[108] E. N. Cerav , Z. Yao , and B. Sun , “Engineered Strategies for Enhancing mRNA Vaccine Stability in Delivery and Storage,” Nanoscale 18, no. 11 (March 2026): 5667–5689, 10.1039/D5NR05189D. 41700648

[109] Q. Li , M. Zeng , W. Lv , J. Ye , and S. Wu , “Current Landscape of Clinical Trials for mRNA‐Based Therapeutics,” Human Vaccines & Immunotherapeutics 22, no. 1 (2026): 2635868, 10.1080/21645515.2026.2635868. PMC12931907 41729563

[110] Y. Shi , M. Shi , Y. Wang , and J. You , “Progress and Prospects of mRNA‐Based Drugs in Pre‐Clinical and Clinical Applications,” Signal Transduction and Targeted Therapy 9, no. 1 (November 2024): 322, 10.1038/s41392-024-02002-z. PMC11564800 39543114

[111] A. Cagigi and K. Loré , “Immune Responses Induced by mRNA Vaccination in Mice, Monkeys and Humans,” Vaccines (Basel) 9, no. 1 (January 2021): 61, 10.3390/vaccines9010061. PMC7831080 33477534

[112] S. Jo , L. Li , C. Thakur , et al., “mRNA Vaccines Engage Unconventional Pathways in CD8 T Cell Priming,” Nature 654, no. 8118 (April 2026): 485–494, 10.1038/s41586-026-10353-6. PMC13089314 41986715

[113] S. Gurung , D. Perocheau , R. Ghosh , S. L. Hart , and J. Baruteau , “Delivering the Message: Translating mRNA Therapy for Liver Inherited Metabolic Diseases,” Journal of Inherited Metabolic Disease 48, no. 5 (September 2025): e70078, 10.1002/jimd.70078. PMC12361847 40826824

[114] T. Cai and X. Q. Yang , “Modified mRNA‐Based Therapeutic Strategies for Myocardial Ischemia‐Reperfusion Injury,” International Journal of Molecular Sciences 27, no. 1 (January 2026): 55, 10.3390/ijms27010055. PMC12785472 41515935

[115] O. Vasileva , O. Zaborova , B. Shmykov , R. Ivanov , and V. Reshetnikov , “Composition of Lipid Nanoparticles for Targeted Delivery: Application to mRNA Therapeutics,” Frontiers in Pharmacology 15 (October 2024): 1466337, 10.3389/fphar.2024.1466337. PMC11537937 39508050

[116] Z. Zhang , J. Du , D. Zhang , R. Han , X. Wu , and Y. Liang , “Research Progress of mRNA Vaccines for Infectious Diseases,” European Journal of Medical Research 30 (August 2025): 792, 10.1186/s40001-025-03060-x. PMC12374293 40847375

[117] S. P. Singh , P. Mundlia , A. Sharma , et al., “A Review on RNA Technologies Renaissance: A Paradigm Shift Shaping the Future of Modern Medicine,” Next Bioengineering 2 (June 2026): 100016, 10.1016/j.nxbio.2026.100016.

[118] B. Yang , J. Liu , Y. Li , and X. Liu , “mRNA Cancer Vaccines: From Pandemic Paradigm to Personalized Oncology Therapeutics,” Cancer Innovation 4, no. 6 (2025): e70041, 10.1002/cai2.70041. PMC12686599 41377933

[119] N. Parvin , T. K. Mandal , and S. W. Joo , “The Impact of COVID‐19 on RNA Therapeutics: A Surge in Lipid Nanoparticles and Alternative Delivery Systems,” Pharmaceutics 16, no. 11 (October 2024): 1366, 10.3390/pharmaceutics16111366. PMC11597542 39598489

[120] J. H. Skerritt , C. Tucek‐Szabo , B. Sutton , and T. Nolan , “The Platform Technology Approach to mRNA Product Development and Regulation,” Vaccines (Basel) 12, no. 5 (May 2024): 528, 10.3390/vaccines12050528. PMC11126020 38793779

[121] J. H. Skerritt , “Considerations for mRNA Product Development, Regulation and Deployment Across the Lifecycle,” Vaccines (Basel) 13, no. 5 (April 2025): 473, 10.3390/vaccines13050473. PMC12116195 40432085

[122] O. Gumusay , J. Callan , and H. S. Rugo , “Immunotherapy Toxicity: Identification and Management,” Breast Cancer Research and Treatment 192, no. 1 (2022): 1–17, 10.1007/s10549-021-06480-5. PMC8841316 35015209

[123] Y. Herdiana , “Bridging the Gap: The Role of Advanced Formulation Strategies in the Clinical Translation of Nanoparticle‐Based Drug Delivery Systems,” International Journal of Nanomedicine 20 (2025): 13039–13053, 10.2147/IJN.S554821. PMC12579833 41185748

[124] F. Stucchi , M. Li , G. Castellano , and F. Cellesi , “Regulatory Framework for Polymer‐Based Nanotherapeutics in Clinical Translation,” Frontiers in Bioengineering and Biotechnology 14 (2026): 1735885, 10.3389/fbioe.2026.1735885. PMC12868300 41647359

[125] S. N. Heendeniya , S. Chen , S. Bhatti , et al., “Beginning of a New Era of Synthetic Messenger RNA Therapeutics: Comprehensive Insights on mRNA Drug Design, Development and Applications,” Experimental Biology and Medicine (Maywood, N.J.) 250 (2025): 10784, 10.3389/ebm.2025.10784. PMC12824530 41586257

[126] X. Hou , T. Zaks , R. Langer , and Y. Dong , “Lipid Nanoparticles for mRNA Delivery,” Nature Reviews Materials 6, no. 12 (2021): 1078–1094, 10.1038/s41578-021-00358-0. PMC8353930 34394960

[127] Y. He , Y. Wang , L. Wang , W. Jiang , and S. Wilhelm , “Understanding Nanoparticle‐Liver Interactions in Nanomedicine,” Expert Opinion on Drug Delivery 21, no. 6 (2024): 829–843, 10.1080/17425247.2024.2375400. PMC11281865 38946471

[128] S. Yan , J. Na , X. Liu , and P. Wu , “Different Targeting Ligands‐Mediated Drug Delivery Systems for Tumor Therapy,” Pharmaceutics 16, no. 2 (2024): 248, 10.3390/pharmaceutics16020248. PMC10893104 38399302

[129] Y. Xu , F. Gong , A. Golubovic , et al., “Rational Design and Modular Synthesis of Biodegradable Ionizable Lipids via the Passerini Reaction for mRNA Delivery,” Proceedings of the National Academy of Sciences of the United States of America 122, no. 5 (2025): e2409572122, 10.1073/pnas.2409572122. PMC11804478 39883839

[130] Y. N. Chen , M. Q. Li , H. J. Zhang , et al., “Nanoparticle‐Based Drug Delivery Systems: A Promising Approach for the Treatment of Liver Fibrosis,” International Journal of Pharmaceutics: X 10 (2025): 100411, 10.1016/j.ijpx.2025.100411. PMC12528922 41113346

[131] A. Ouranidis , T. Vavilis , E. Mandala , et al., “mRNA Therapeutic Modalities Design, Formulation and Manufacturing Under Pharma 4.0 Principles,” Biomedicines 10, no. 1 (2021): 50, 10.3390/biomedicines10010050. PMC8773365 35052730

[132] A. I. Paremskaia , A. A. Kogan , A. Murashkina , et al., “Codon‐Optimization in Gene Therapy: Promises, Prospects and Challenges,” Frontiers in Bioengineering and Biotechnology 12 (2024): 1371596, 10.3389/fbioe.2024.1371596. PMC11007035 38605988

[133] M. J. Moss , L. M. Chamness , and P. L. Clark , “The Effects of Codon Usage on Protein Structure and Folding,” Annual Review of Biophysics 53, no. 1 (2024): 87–108, 10.1146/annurev-biophys-030722-020555. PMC11227313 38134335

[134] E. A. Demissie , S. Y. Park , J. H. Moon , and D. Y. Lee , “Comparative Analysis of Codon Optimization Tools: Advancing Toward a Multi‐Criteria Framework for Synthetic Gene Design,” Journal of Microbiology and Biotechnology 35 (2025): e2411066, 10.4014/jmb.2411.11066. PMC12010093 40223268

[135] A. Muslimov , V. Tereshchenko , D. Shevyrev , et al., “The Dual Role of the Innate Immune System in the Effectiveness of mRNA Therapeutics,” International Journal of Molecular Sciences 24, no. 19 (2023): 14820, 10.3390/ijms241914820. PMC10573212 37834268

[136] X. Piao , V. Yadav , E. Wang , et al., “Double‐Stranded RNA Reduction by Chaotropic Agents During In Vitro Transcription of Messenger RNA,” Molecular Therapy. Nucleic Acids 29 (2022): 618, 10.1016/j.omtn.2022.08.001. PMC9421179 36090758

[137] Y. Eralp , “Application of mRNA Technology in Cancer Therapeutics,” Vaccines (Basel) 10, no. 8 (2022): 1262, 10.3390/vaccines10081262. PMC9415393 36016150

[138] C. Qiu , Y. Wu , Q. Shi , et al., “Advanced Strategies for Nucleic Acids and Small‐Molecular Drugs in Combined Anticancer Therapy,” International Journal of Biological Sciences 19, no. 3 (2023): 789–810, 10.7150/ijbs.79328. PMC9910002 36778126

[139] B. Kong , Y. Kim , E. H. Kim , J. S. Suk , and Y. Yang , “mRNA: A Promising Platform for Cancer Immunotherapy,” Advanced Drug Delivery Reviews 199 (2023): 114993, 10.1016/j.addr.2023.114993. PMC11797636 37414361

[140] A. Afzal , M. H. Abbasi , S. Ahmad , N. Sheikh , and M. B. Khawar , “Current Trends in Messenger RNA Technology for Cancer Therapeutics,” Biomaterials Research 29 (2029): 0178, 10.34133/bmr.0178. PMC11978394 40207255

[141] A. Singh , M. Tekade , S. Nagaraja , A. Bharti , and R. K. Tekade , “Advancements in RNA‐Based Therapies From Bench to Bedside,” npj Drug Discovery 3, no. 1 (2026): 4, 10.1038/s44386-025-00037-y. PMC13252867 42380588

[142] L. Zhong , L. Tao , Z. Zhai , et al., “The Stability Landscape of mRNA Vaccines: Challenges and Mitigation Strategies,” Vaccine 79 (2026): 128515, 10.1016/j.vaccine.2026.128515. 41880821

[143] E. Monfrini , G. Baso , D. Ronchi , et al., “Unleashing the Potential of mRNA Therapeutics for Inherited Neurological Diseases,” Brain 147, no. 9 (2024): 2934–2945, 10.1093/brain/awae135. PMC11969220 38662782

[144] M. F. S. Fernandes , A. Alexe , O. Apara , et al., “Post‐Approval Activities Providing Data on the Safety of Medication Use During Pregnancy and Lactation: A Transcelerate Perspective,” Therapeutic Innovation & Regulatory Science 59, no. 3 (2025): 527, 10.1007/s43441-025-00764-4. PMC12018607 40091001

[145] C. J. A. Nowak , S. Liu , R. J. Falconer , and L. Gerstweiler , “Process and Analytical Strategies for the Safe Production of mRNA Vaccines and Therapeutics,” Molecular Biology Reports 53, no. 1 (2026): 306, 10.1007/s11033-026-11455-0. PMC12819531 41557011

[146] M. Youssef , C. Hitti , J. Puppin Chaves Fulber , and A. A. Kamen , “Enabling mRNA Therapeutics: Current Landscape and Challenges in Manufacturing,” Biomolecules 13, no. 10 (2023): 1497, 10.3390/biom13101497. PMC10604719 37892179

[147] C. Chandipwisa , A. Shimilimo , O. Uwishema , and H. Onyeaka , “The Current Landscape and Future Prospects of Vaccine Manufacturing in Africa: Challenges, Innovations, and Opportunities: A Narrative Review,” Health Science Reports 9, no. 3 (2026): e71926, 10.1002/hsr2.71926. PMC12949823 41773217

[148] M. Moschioni , R. A. Siraji , R. Dissard , et al., “mRNA Vaccines and Therapeutics Beyond COVID‐19: A Review of the Global Clinical Development Landscape, Low‐ and Middle‐Income Countries Involvement and Relevance to Their Contexts,” Human Vaccines & Immunotherapeutics 22, no. 1 (2026): 2628424, 10.1080/21645515.2026.2628424. PMC12962614 41781347

[149] A. D. J. Lopes De Abreu , C. A. M. Mpande , Y. Song , M. W. Nicholson , C. Nannei , and M. Friede , “Unpacking the mRNA Supply Chain: Challenges and Opportunities for Global Health,” Vaccines (Basel) 14, no. 4 (2026): 324, 10.3390/vaccines14040324. PMC13119685 42042800

[150] S. Akkineni , M. Gulani , S. A. Kouzi , M. J. D'Souza , and M. N. Uddin , “Delivery of mRNA Therapeutics Beyond Infectious Diseases: Design Innovations and Applications in Oncology, Cardiovascular, and Rare Genetic Diseases,” Pharmaceuticals (Basel) 19, no. 5 (2026): 663, 10.3390/ph19050663. PMC13209915 42198337

[151] S. M. Iqbal , A. M. Rosen , D. Edwards , et al., “Opportunities and Challenges to Implementing mRNA‐Based Vaccines and Medicines: Lessons From COVID‐19,” Frontiers in Public Health 12 (2024): 1429265, 10.3389/fpubh.2024.1429265. PMC11340501 39175908

[152] R. Srivastava , “AI‐Powered Mapping of Tumor Immunity for Optimized mRNA Vaccine Engineering,” Frontiers in Oncology 16 (2026): 1766201, 10.3389/fonc.2026.1766201. PMC12992067 41853314

[153] H. Kong , “Advances in Personalized Cancer Vaccine Development: AI Applications From Neoantigen Discovery to mRNA Formulation,” BioChem 5, no. 2 (2025): 5, 10.3390/biochem5020005.

[154] A. A. Belaidi , D. L. Furtado , F. Alves , R. M. Nisbet , S. Ayton , and A. Bush , “Engineering mRNA‐LNP Medicines for the Ageing Brain: Opportunities and Challenges for Neurodegenerative Diseases,” [Internet], Authorea, accessed April 27, 2026, https://www.authorea.com/users/977189/articles/1344362-engineering-mrna-lnp-medicines-for-the-ageing-brain-opportunities-and-challenges-for-neurodegenerative-diseases. 10.1002/exp2.70223 PMC13551305 42713005

[155] V. Bhati , S. Prasad , and A. Kabra , “RNA‐Based Therapies for Neurodegenerative Disease: Targeting Molecular Mechanisms for Disease Modification,” Molecular and Cellular Neuroscience 133 (2025): 104010, 10.1016/j.mcn.2025.104010. 40340000

[156] S. Patel , A. Athirasala , P. P. Menezes , et al., “Messenger RNA Delivery for Tissue Engineering and Regenerative Medicine Applications,” Tissue Engineering. Part A 25, no. 1–2 (2019): 91–112, 10.1089/ten.tea.2017.0444. PMC6352544 29661055

[157] D. Xue , Y. Zhang , A. Shen , T. Tang , B. Liu , and L. Lv , “mRNA Therapy: A New Frontier in Regenerative Medicine,” Interdisciplinary Medicine 3 (2025): e20240054, 10.1002/INMD.20240054.

[158] A. Paramasivan , “The Future of Personalized Medicine AI‐Driven Solutions in Drug Discovery and Patient Care,” Journal of Science and Engineering Research 8 (2021): 256–263, 10.5281/zenodo.14554637.

[159] S. Imani , X. Li , K. Chen , et al., “Computational Biology and Artificial Intelligence in mRNA Vaccine Design for Cancer Immunotherapy,” Frontiers in Cellular and Infection Microbiology 14 (2025): 1501010, 10.3389/fcimb.2024.1501010. PMC11788159 39902185

